# Rational molecular engineering of cyclopentadithiophene-bridged D-A-π-A sensitizers combining high photovoltaic efficiency with rapid dye adsorption

**DOI:** 10.1038/srep11330

**Published:** 2015-06-11

**Authors:** Qipeng Chai, Wenqin Li, Jingchuan Liu, Zhiyuan Geng, He Tian, Wei-hong Zhu

**Affiliations:** 1 Shanghai Key Laboratory of Functional Materials Chemistry, Key Laboratory for Advanced Materials and Institute of Fine Chemicals, Collaborative Innovation Center for Coal Based Energy (i-CCE), East China University of Science and Technology, Shanghai 200237, P. R. China; 2 School of Urban Development and Environmental Engineering, Shanghai Second Polytechnic University, Shanghai 201209, P. R. China; 3 Gansu Key Laboratory of Polymer Materials, College of Chemistry and Chemical Engineering, Key Laboratory of Eco-environment-related Polymer Materials, Ministry of Education, Northwest Normal University, Lanzhou, Gansu 730070, P. R. China

## Abstract

Dye-sensitized solar cell (DSSC) is considered as a feasible route to the clean and renewable energy conversion technique. The commercial application requires further enhancements on photovoltaic efficiency and simplification on the device fabrication. For avoiding the unpreferable trade-off between photocurrent (*J*_SC_) and photovoltage (*V*_OC_), here we report the molecular engineering and comprehensive photovoltaic characterization of three cyclopentadithiophene-bridged D-A-π-A motif sensitizers with a change in donor group. We make a careful choice on the donor and conjugation bridge for synergistically increasing *J*_SC_ and *V*_OC_. Comparing with the reference dye **WS-2**, the photovoltaic efficiency with the single component dye of **WS-51** increases by 18%, among one of the rare examples in pure metal-free organic dyes exceeding 10% in combination with traditional iodine redox couples. Moreover, **WS-51** exhibits several prominent merits on potentially scale-up industrial application: i) facile synthetic route to target molecule, ii) simple dipping procedure without requirement of co-sensitization, and iii) rapid dye adsorption capability.

Dye-sensitized solar cells (DSSCs) have aroused great enthusiasm as an alternative photoelectric conversion device^1^,^2^, with extensive efforts to pursuing high photovoltaic efficiency and low cost for their commercial attractiveness^3^,^4^,^5^,^6^,^7^,^8^,^9^. In DSSCs, sunlight is harvested by dye sensitizers that are attached onto the surface of a wide band gap semiconductor oxide (typically TiO_2_). Generally, organic sensitizers govern photon harvesting and electron conduction inside nanoporous TiO_2_ electrode, being one of the decisive factors to high photon-conversion-efficiency (PCE). Organic sensitizers usually have a donor-π-bridge-acceptor (D−π−A) feature, with a characteristic intramolecular charge transfer (ICT) band that harvests sunlight for photon-to-electron conversion. However, the absorption bands for most pure organic dyes based on D−π−A motif always fall in short wavelength region (less than 500 nm)^3^,^4^. Moreover, due to the deprotonation effect, conventional D−π−A sensitizers bring forth large blue-shifts when anchored onto TiO_2_ photoanodes, leading to a great loss in photocurrent generation.

To minimize the limitation, a concept of D−A−π−A motif was proposed for designing a new generation of efficient and stable organic sensitizers, in which an auxiliary electron-withdrawing unit was incorporated into the conjugated bridge to facilitate electron transfer from the donor to the acceptor^10^,^11^,^12^,^13^,^14^,^15^,^16^,^17^,^18^,^19^,^20^,^21^,^22^,^23^,^24^,^25^. As an electron deficient unit, the incorporated additional unit can efficiently modulate the energy levels, absorption bands, and function as a great relief of the blue shifts when the dye molecules anchored on TiO_2_ anode, which is beneficial to the photocurrent generation with extending the incident photon-to-electron conversion efficiency (IPCE) onset^10^. For instance, our group systematically studied a series of 2,1,3-benzothiadiazole (BTD)-based sensitizers based on D−A−π−A motif, showing high PCE of 8.7% (**WS-2**) and 9.0% (**WS-9**) with the iodine redox couples^11^,^13^. However, the photovoltage in these BTD based D−A−π−A sensitizers is always unsatisfactory due to the serious electron recombination. The known **WS-2** endowed a moderate photovoltage in the range of 580–650 mV^11^,^12^, which critically limits the photovoltaic efficiency to a great extent.

As well known, the presence of long alkyl chains on conjugated bridge could efficiently decrease the intermolecular interactions and retard the electron recombination, thus resulting in an obvious improvement of *V*_OC_^14^,^26^. As an effective building block, 4*H*-cyclopenta[2,1-*b*:3,4-*b*’]dithiophene (CPDT) has been exploited for constructing the π−conjugated skeleton of organic sensitizers due to its excellent co-planarity and electron donating capability^27^,^28^,^29^. Moreover, the long alkyl chains can be feasibly introduced at the bridging carbon atoms of CPDT. Herein, we further replace the thiophene moiety (in the D−A−π−A motif of **WS-2**) with the enlarged segment dioctyl-substituted CPDT for the sake of extending IPCE response and attenuating the interfacial interactions. The three CPDT-bridged D-A-π-A sensitizers (Fig. 1) with a change in donor group were rationally designed. Comparing with the reference dye **WS-2**, the CPDT-based corresponding dye **WS-51** exhibits a panchromatic profile with a synergistic enhancement in *J*_SC_ and *V*_OC_, achieving an impressive 10.1% PCE without requirement of co-sensitization. More interestingly, instead of normal long term adsorption (>12 h), **WS-51** shows a rapid adsorption property and reaches a PCE over 9% after only initial 2 h dye adsorption. As a consequence, dye **WS-51** exhibits several prominent merits, such as convenient synthesis, simple dipping procedure, no necessary co-sensitization, and rapid dye adsorption, which are highly preferable for potentially industrial scale-up application owing to its high efficiency and time-saving dye adsorption.

## Results

### Molecular design and synthesis

**Figure 2:**
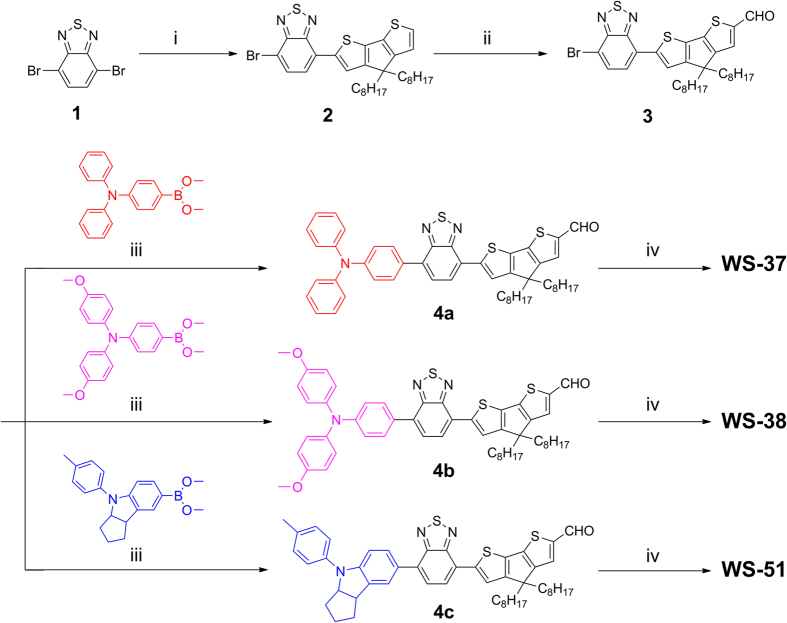
Synthetic route to WS-37, WS-38 and WS-51. Reaction conditions: i) Pd(PPh_3_)_4_, 2 M K_2_CO_3_ aqueous solution, THF, Ar, 90 ºC, ii) POCl_3_, DMF, 25 ºC, iii) Pd(PPh_3_)_4_, 2 M K_2_CO_3_ aqueous solution, THF, Ar, 90 ºC, and iv) piperidine, acetonitrile, Ar, 90 ºC.

As an excellent co-planarity, the enlarged conjugation CPDT was utilized instead of thiophene to effectuate the red-shift and enhancement in ICT absorption band for extending light-harvesting. Moreover, the introduction of long alkyl chains would also attenuate the interfacial recombination, for achieving both gains in *J*_SC_ and *V*_OC_. Several classical donor groups were incorporated in molecular skeleton to modulate the orbital levels for balancing the thermodynamic force between electron injection and dye regeneration. As illustrated in Fig. 2, we utilized the facile starting material of 4,7-dibromobenzo[*c*][1,2,5]thiadiazole to develop these sensitizers. Dioctyl-substituted CPDT moiety was attached to the additional unit of BTD via Suzuki reaction and afforded a monosubstituted product **2**. Then the formylation gave aldehyde **3** under room temperature. Subsequently, the donor groups and the framework **3** coupled together via Suzuki reaction to give the sensitizer precursors **4**, which were eventually converted to the target dyes (**WS-37**, **WS-38** and **WS-51**) by reflux for 8 h in the presence of piperidine and acetonitrile. All the dye sensitizers are dark purple in solid state, and exhibit purple red to dark purple in solutions with gradually enhanced electron donating capability in the donor units.

### Red-shift and molar extinction coefficient enhancement in absorption band

**Figure 3:**
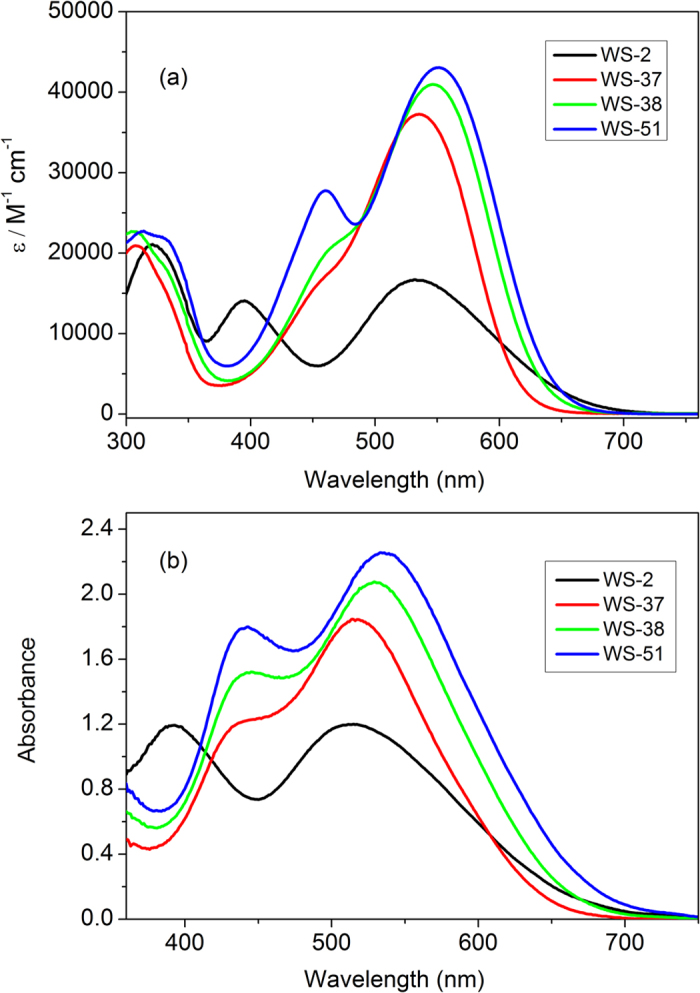
Absorption spectra of sensitizers WS-37, WS-38 and WS-51 and reference dye WS-2 in mixed solution (CHCl3:CH3OH, v/v = 4:1 , **a**) and on 3 μmTiO_2_ films (**b**).

The absorption spectra of dyes (**WS-37**, **WS-38** and **WS-51**) in mixed solution (CHCl_3_:CH_3_OH, v/v = 4:1) and on TiO_2_ films were preliminary studied (Fig. 3 and Table 1). In similar with **WS-2**, the three BTD based D−A−π−A sensitizers exhibit three major electronic absorption bands: i) the π–π* electron transitions in UV region (near 300 nm), ii) the ICT band in visible region (around 540 nm), and iii) the additional absorption band or shoulder from subordinate orbital transition (near 460 nm, DFT calculation, Supplementary Table S1). The direct comparison between **WS-2** and **WS-51** highlights the effect of CPDT conjugation bridge on the ICT absorption band as well as molar extinction coefficients. Compared with **WS-2** (*λ*_max_ = 533 nm), the larger co-planarity of CPDT unit in the skeleton of **WS-51** results in a red-shift by 18 nm. Moreover, **WS-51** exhibits 1.6-fold higher molar extinction coefficient (*ε* = 43000 M^−1^ cm^−1^) than **WS-2** (*ε* = 16700 M^−1^ cm^−1^, Table 1). The tendency is well consistent with the calculated band gap and oscillator strength (Supplementary Table S1). Moreover, in spite of giving the similar absorption profile (Fig. 3), the gradually enhanced electron donating capability can also red shift the absorption band, from triphenylamine unit (**WS-37**, *λ*_max_ = 536 nm), dialkoxy-substituted triphenylamine unit (**WS-38**, *λ*_max_ = 546 nm) to indoline unit (**WS-51**, *λ*_max_ = 551 nm). In this sense, the indoline unit in **WS-51** is stronger than the traditional donor unit of tripenylamine unit^11^,^16^. Additionally, the blue shifts on TiO_2_ films for the BTD based D−A−π−A sensitizers are all around 20 nm (Table 1), much less than that of conventional D−π−A motif, highlighting the additional electron-withdrawing effect of BTD on the weakening influence by deprotonation.

### Electrochemical data and DTF simulation

To further determine the energy level position of frontier orbitals, cyclic voltammetry was performed in CH_2_Cl_2_ with tetra-*n*-butylammonium hexafluorophosphate (TBAHFP) as a supporting electrolyte. The formal oxidation potentials are corresponding to the HOMO levels, appearing at 0.82 and 0.90 V (*vs* NHE) for **WS-51** and **WS-2**, respectively. The 0.08 V upshift can be ascribed to the contribution of a higher electron-rich effect of CPDT relative to thiophene moiety. Moreover, all the CPDT-based dyes exhibit two reversible oxidation waves (Supplementary Fig. S1). The resulting formal oxidation potentials are found at 1.05, 0.85 and 0.82 V for **WS-37**, **WS-38** and **WS-51**, respectively. Here the uplifted HOMO levels are in accordance with the increasing electron donating ability for each donor group. Derived from the absorption thresholds on TiO_2_ films, the resulting LUMO levels for **WS-2**, **WS-37**, **WS-38** and **WS-51** are −1.00, −0.91, −1.04 and −1.01 V, respectively. Given the chemical potential of iodine redox couples (0.4 V *vs* NHE) and the conduction band position of TiO_2_ (−0.5 V), there exists sufficient thermodynamic force for the occurrence of dye regeneration and electron injection^30^,^31^.

**Figure 4:**
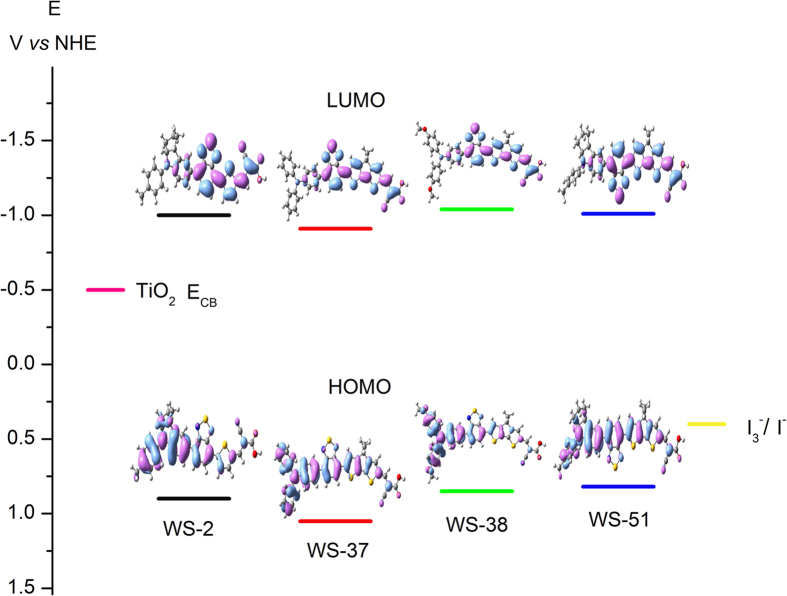
Calculated frontier molecular orbitals (HOMO and LUMO) of sensitizers WS-2, WS-37, WS-38 and WS-51.

The electron distribution in the frontier orbitals for these sensitizers is presented in Fig. 4, and their optimized geometrical configurations are shown in Supplementary Fig. S2. The HOMO electrons in all these four sensitizers are delocalized throughout the entire framework, indicating that they can be treated as highly conjugated configurations with excellent electron transfer channels for electron injection. The LUMO electrons are predominantly distributed on the BTD-CPDT-anchor unit. Obviously, the good overlap between the HOMO−LUMO orbital can facilitate the electron migration from donor to the anchor unit, then to the conduction band of semiconductor TiO_2_. Besides, the small dihedral angles between BTD and CPDT (Supplementary Fig. S2) emphasize the convenience in electron flow through the entire skeleton.

### Synergistic enhancement of *J*_SC_ and *V*_OC_

**Figure 5:**
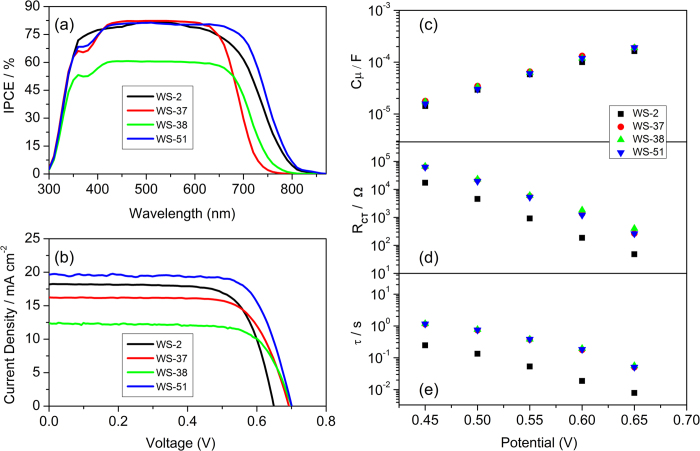
IPCE action spectra (**a**), current−voltage characteristics (**b**), plots of cell capacitance (*C*_μ_, **c**), interface charge transfer resistance (*R*_CT_, **d**) and calculated electron lifetime (*τ*, **e**) under a series potential bias of DSSCs based on sensitizers **WS-2**, **WS-37**, **WS-38** and **WS-51** (dye bath solvent: CHCl_3_:C_2_H_5_OH, v/v = 1:1, dipping time: 12 h)

Generally, the photovoltaic efficiency can be optimized from the enhancement of *J*_SC_ and *V*_OC_. However, the unpreferable trade-off between *J*_SC_ and *V*_OC_ is always observed. For instance, the broad spectral response with high photocurrent has been easily achieved in BTD-based sensitizers, while their *V*_OC_ is always limited to 670 mV^11^,^12^. In contrast, the high *V*_OC_ is easy to realize in benzotriazole-based sensitizers but their photocurrent becomes discouraging to some extent^14^,^15^. As well known, the quantum conversion yield in response region and the spectrum coverage range determine the generation of photocurrent density. Our motivation is to look for a sensitizer holding the capability of panchromatic coverage and particularly efficient electron conversion, keeping the synergistic enhancement of *J*_SC_ and *V*_OC_. Fig. 5a shows the IPCE curves as a function of excitation wavelength for the developed sensitizers. Notably, the IPCE onsets are well consistent with the corresponding electron donating ability, that is, indoline (**WS-51)** > dimethoxy-substituted triphenylamine (**WS-38**) > triphenylamine (**WS-37**). Especially, all the CPDT-based devices have small notches around 400 nm, which is attributed to the weak absorbance in this region. As a matter of fact, **WS-51** exhibited the extensive plateau IPCE response, above 80% in the entire visible region of 400**–**700 nm. Indeed, among the four dye sensitizers, the predominance from both the CPDT conjugation bridge and indoline unit in **WS-51** realized a broadest spectral response, thus presenting a high current density (19.69 ± 0.09 mA cm^−2^) with respect to **WS-2** (18.24 ± 0.24 mA cm^−2^). Here the integrals of IPCE curves are in good agreement with the measured current density, showing less than 5.2% error. However, when compared with **WS-37** and **WS-51**, **WS-38** displayed the worst IPCE response value with around 60% plateau in the visible region, resulting in a low *J*_SC_ (12.32 mA cm^−2^).

Besides *J*_SC_, the different donor parts in **WS-37**, **WS-38** and **WS-51** have little impacts on *V*_OC_ (690–700 mV). Consequently, we shed the improvement of photovoltage (*V*_OC_) on the facile introduction of hydrophobic long alkyl group on CPDT as anti-aggregation chain. The reference sensitizer **WS-2** displayed a moderate photovoltage below 650 mV, which critically limited the photovoltaic efficiency to a great extent. Interestingly, the *V*_OC_ was increased by around 50 mV when simply changing the conjugated bridge from thiophene to CPDT unit. Compared with **WS-2**, the incorporation of dioctyl-substituted CPDT in **WS-51** can offer the possibility to reduce the intermolecular interactions or block molecular aggregation. In fact, the negative effect upon the addition of a co-adsorbent CDCA (Table 2) is suggestive that the steric two octyl chains can efficiently suppress the formation of undesirable dye aggregates. Considering the limitation to the relatively low *V*_OC_ in **WS-2**, the rational molecular strategy with the dialkyl substituted CPDT conjugated bridge and indoline unit in **WS-51** can guarantee the balance of *J*_SC_ and *V*_OC_, especially pursuing the goal of outstanding device efficiency. As a result, under standard AM 1.5 conditions, **WS-51**-based solar cells realized an outstanding photovoltaic efficiency of 10.08 ± 0.05% (*J*_SC_ = 19.69 mA cm^−2^, *V*_OC_ = 700 mV, *FF* = 0.73). In this way, the synergistic uplift from both *J*_SC_ and *V*_OC_ in **WS-51** lead to an increase by 18.7% in photovoltaic efficiency with respect to **WS-2**. Additionally, the power conversion efficiency of **WS-51** can remain above 90% of initial value under one-sun illumination after 1000 h, indicative of good sensitizer stability.

Generally, the *V*_OC_ is defined as the potential difference between Femi level of TiO_2_ (*E*_Fn_) and the chemical potential of redox mediators in the electrolyte. Due to the identical redox species in the test, the *V*_OC_ is determined by the position of conduction band of TiO_2_ and the electron density in the TiO_2_ nanoparticles^32^,^33^,^34^,^35^. To identify the position of TiO_2_ conduction band, we fitted the cell capacitance (*C*_μ_) responses under a series bias potential, determined from the typical electrochemical impedance spectroscopy (EIS). In these solar cells sensitized with these four dyes, the logarithm of *C*_μ_ was enhanced at the almost identical slope, showing a linear increase with the given bias potential (Fig. 5c). Obviously, the observed similar *C*_μ_ value at fixed potential is indicative of no essential influence on the conduction band of TiO_2_. In other words, the values of *V*_OC_ is directly correlated with the electron density in TiO_2_. Compared with **WS-2**, the congener **WS-51** exhibited an obvious uplift of the charge transfer resistance (*R*_CT_) under a series bias potential. In particular, the *R*_CT_ of **WS-51**-based device was 6-fold as that for **WS-2** under 0.65 V (Fig. 5d). Obviously, **WS-51** is more efficient for inhibition of the interfacial electron recombination process^36^,^37^,^38^. Also, **WS-37** and **WS-38** displayed a comparable *R*_CT_ and calculated electron lifetime (*τ*, Fig. 5e) with **WS-51**. Obviously, the different donor parts in **WS-37**, **WS-38** and **WS-51** have a negligible effect on the interfacial interactions or *V*_OC_. As a consequence, compared with **WS-2**, it is the dioctyl-substituted CPDT bridge that plays a major role in the observed synergistic enhancement of *J*_SC_ and *V*_OC_, specifically resulting in a 18.7% increase in photovoltaic efficiency of **WS-51**.

### Rapid dye uptake

**Figure 6:**
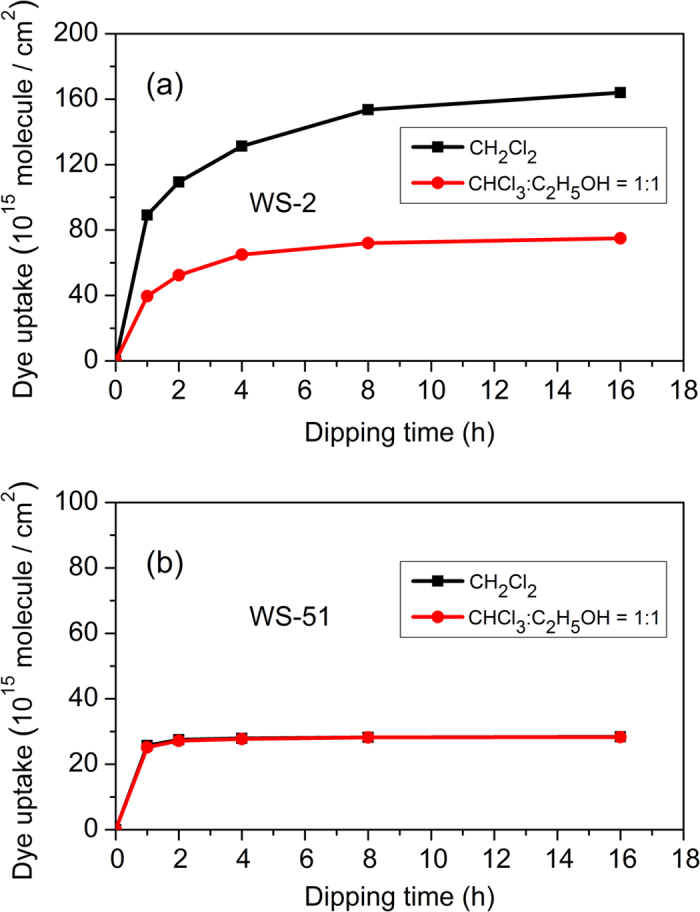
Absorption profiles of **WS-2** (**a**) and **WS-51** (**b**) on TiO_2_ photoanodes under different dye bath system.

In our previous work, CH_2_Cl_2_ was chosen as dye bath solvent for **WS-2**. In a preliminary test, the photovoltaic performance of **WS-2** is not satisfying due to the serious aggregation. Therefore, chenodeoxycholic acid (CDCA) was used as a co-adsorbent for the consequent optimization of the devices^11^,^12^,^13^. Herein, we adopted a binary solvent system (CHCl_3_:C_2_H_5_OH, v/v = 1:1) to optimize the DSSC performance. Meanwhile, the dye uptake experiments were attentively performed to gain insight into the origin of the remarkable difference on photovoltaic performances between the two kinds of dye bath solvents. Fig. 6 depicted the dye adsorption profiles as a function of time for **WS-2** and **WS-51**. In both cases, the dye uptake amounts were increased significantly in initial 2 h. Afterwards, the uptrends slowed down gradually, and reached plateau at a certain time (>8 h). The dye uptake amount for **WS-2** in CH_2_Cl_2_ was 2-fold of that in binary solvent system after complete adsorption. In sharp contrast, **WS-51** did not show any difference in either CH_2_Cl_2_ or the binary solvents (CHCl_3_:C_2_H_5_OH). That is, the dye loading amount of **WS-51** in the two kinds of dye bath solvents was fundamentally equal at any dye soaking time. In particular, over 90% dye molecules of **WS-51** were binding onto TiO_2_ nanoparticles after initial 2 h dye adsorption. Moreover, FTIR spectroscopic analyses confirmed the same binding mode in the two above-mentioned dye bath solvents, along with the identical peak locations: carboxylate asymmetric stretching vibration (−COO^−^_*as*_) around 1612 cm^−1^ and symmetric stretching vibration (−COO^−^_*s*_) around 1400 cm^−1^ (Supplementary Fig. S3).

**Figure 7:**
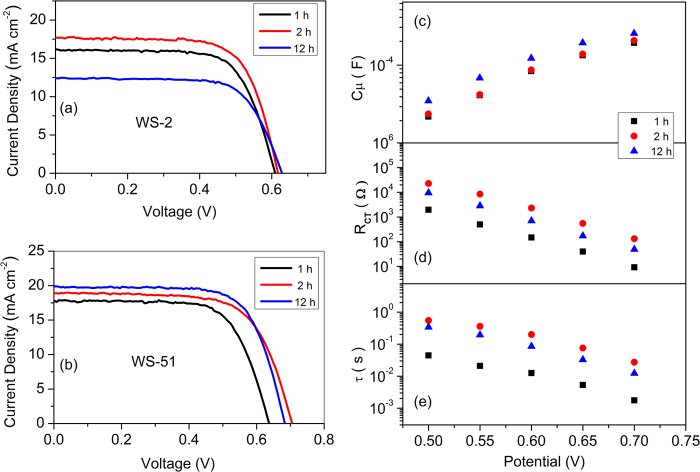
Current−voltage characteristics of **WS-2** (**a**), **WS-51** (**b**), plots of cell capacitance (*C*_μ_, **c**), interface charge transfer resistance (*R*_CT_, **d**) and calculated electron lifetime (*τ*, **e**) under a series potential bias of DSSCs based on **WS-51** under a series dye soaking time (1, 2 and 12 h) with dye bath solvent of CH_2_Cl_2_

According to Fig. 6, it is obvious that the device performance may have a close relationship with the dye uptake amount for a designated dye in a given dye bath solvent. In view of the sharp increase in dye adsorption amount during the initial dipping time, we measured the DSSC performance with different dye adsorption amount. When using CH_2_Cl_2_ as dye bath solvent, the photovoltaic efficiency around 7% was obtained with 1 h adsorption of **WS-2**, and further improvement to 7.6% was reached after another 1 h soaking (Fig. 7a, Table 3). However, when the dipping time was delayed to 12 h, the *J*_SC_ was decreased sharply from 17.67 to 12.43 mA cm^−2^, resulting in a low photovoltaic efficiency of 5.50%. The decrease in photovoltaic performance implies that the serious aggregation for **WS-2** would take place during the time from 2 h to 12 h. That is why we have to make use of chenodeoxycholic acid (CDCA) as a co-adsorbent for the performance optimization^11^,^12^,^13^. In contrast with **WS-2**, the photovoltaic efficiency of **WS-51** was enhanced with the increasing dipping time (Fig. 7b, Table 3). In fact, the overall performance point of inflection was not observed, even without the requirement of co-sensitization, which might be attributed to the existing long dioctyl chain in CPDT unit. An impressive conversion efficiency of 9% was obtained with only 2 h adsorption of **WS-51**, around 94% of that in 12 h common dipping condition. As a consequence, the rapid dye uptake of **WS-51** becomes very attractive, which may avoid the tedious time-consuming process in development of large scale commercial DSSCs.

To further scrutinize the different *V*_OC_ of **WS-51** under various dye-soaking time, the EIS analyses were also performed. In initial 1 h, the dye coverage is not sufficient to some extent, resulting in a relatively low light harvesting efficiency and a moderate photocurrent density. Besides, the more unoccupied sites on TiO_2_ nanoparticles may facilitate the recombination between the injected electrons and the redox species, thus presenting a relatively low *V*_OC_ (637 mV). With subsequent 1 h dipping, no relative shifts of conduction band in TiO_2_ were observed (Fig. 7c). Whereas, the charge transfer resistances displayed significant enhancements (Fig. 7d) due to the consequent coverage of the unoccupied sites, which is more efficient to retard the deleterious electron recombination. With a 12 h common dipping time, the devices indicated a relative downward CB compared with those for 1 h and 2 h conditions. Meanwhile, the *R*_CT_ values of 12 h under a series bias potential were also between those of 1 h and 2 h. As a consequence, the combined effect explains the trend of the electron lifetime (*τ*_2h_ > *τ*_12h_ > *τ*_1h_, Fig. 7e), which is well consistent with the *V*_OC_ change tendency from 637, 705 to 683 mV (Table 3) with dipping time for 1, 2 and 12 h, respectively.

## Discussion

The photovoltaic efficiency can be optimized from the enhancement of *J*_SC_ and *V*_OC_. However, the unpreferable trade-off between *J*_SC_ and *V*_OC_ is always observed. Our main motivation is to look for a sensitizer holding the capability of panchromatic coverage and particularly efficient electron conversion. In this work, we make a rational molecular design on the component in the sensitizer framework for the sake of keeping the synergistic enhancement of *J*_SC_ and *V*_OC_^39^,^40^,^41^: i) A powerful electron donating group of indoline pushes the photoexcitation electron vigorously through the molecular channel to the internal TiO_2_ nanoparticles, ii) the auxiliary electron-withdrawing unit BTD extends the spectral response and weakens the blue shift on TiO_2_ films, and iii) the enlarged π-conjugation bridge of CPDT with long alkyl chain further extends the response wavelength, enhances the light harvesting capability, and blocking aggregation for free co-sensitization.

We modulated the electron donating group and conjugation bridge in three cyclopentadithiophene-bridged D-A-π-A motif sensitizer, for balancing dye regeneration and electron injection. The device performance indicates that the donor groups have a determined influence on the photocurrent but with little impact on photovoltage. The combination of a powerful electron donating unit indoline, a strong electron-deficient moiety BTD and an enlarged π-conjugation CPDT brings forth a sensitizer named **WS-51** with several characteristics, such as a panchromatic absorption profile, no requirement of co-sensitization, and rapid dye adsorption capability. **WS-51** achieved an impressive device efficiency up to 10.1%, which is very rare in single organic dye working with iodine electrolyte. Moreover, **WS-51** presents a rapid dye adsorption, and the photovoltaic device efficiency in a 2 h dipping can reach 94% that for common 12 h soaking, effectively avoiding the time-consuming method for industrial application in the future. As demonstrated, **WS-51** is a promising dye sensitizer in DSSCs, especially in consideration of the commercialization of DSSCs owing to its facile synthesis, high efficiency and rapid dye adsorption.

## Methods

### General

All materials and detailed synthetic procedure are collected in Supplementary Information. ^1^H and ^13^C NMR and HRMS were recorded on Bruker 400 and Waters ESI mass spectroscopy, respectively, for the characterization of intermediates and target molecules. The UV-vis absorption spectra were obtained with CARY 100 spectroscopy, and infrared (IR) spectra were performed using Nicolet 380 FTIR spectrometer with dye-loaded TiO_2_ powder. Cyclic voltammetry was measured with a three-electrode system, a calomel electrode in saturated KCl solution as reference electrode, Pt as working electrode, and a Pt wire as counter electrode, respectively. The redox couple of Fc/Fc^+^ was measured as external standard.

### Device fabrication and photovoltaic characterization

The photoelectrode was fabricated by repeating screen printing process with commercial colloidal paste (Dyesol 18NR-T) layer (12 μm) and scattering layer (4 μm), respectively. Afterwards, the TiO_2_ films were heated gradually under an air flow at 325 °C for 5 min, 375 °C for 5 min, 450 °C for 15 min, and 500 °C for 15 min. Prior to dye adsorption, the TiO_2_ films were post treated by 0.04 M TiCl_4_ solution to increase the surface area and improve the connectivity of the nanoparticles. Subsequently, the photoanodes sintered once again and cooled to room temperature. Then they were immersed into a binary solvent system (CHCl_3_:C_2_H_5_OH = 1:1) or CH_2_Cl_2_ with sensitizers (3 × 10^−4^ M), respectively. For the counter electrode, the H_2_PtCl_6_ in 2-propanol solution presented a uniform distribution on FTO glass by spin coating method, and the cathode was heated under 400 °C for deposition of platinum. Eventually, the two electrodes were sealed with thermoplastic Surlyn, and an electrolyte solution was introduced through one hole in the counter electrode to finish the sandwiches type-solar cells. The electrolyte is composed of 0.6 M DMPII, 0.1 M LiI, 0.05 M I_2_, and 0.5 M TBP in acetonitrile. The photocurrent-voltage (*I*-*V*) curves were measured under AM1.5G simulated solar light by illuminating the cell through the FTO substrate from the photoanode side. The incident photon-to-charge carrier efficiencies (IPCEs) were obtained on a Newport-74125system (Newport instruments). Electronic impedance spectra (EIS) measurements was performed with an impedance analyzer (Solartron Analytical, 1255B) using DSSC devices under 20 °C in the dark. The applied frequency range was 10^−1^–10^5^ Hz, and the magnitude of the sinusoidal perturbations was 5 mV. The bias potential varied between 450 and 650 mV, or from 500 to 700 mV, with about 50 mV progressive increase, and the spectra was characterized with Z-View software.

## Additional Information

**How to cite this article**: Chai, Q. *et al.* Rational molecular engineering of cyclopentadithiophene-bridged D-A-π-A sensitizers combining high photovoltaic efficiency with rapid dye adsorption. *Sci. Rep.* **5**, 11330; doi: 10.1038/srep11330 (2015).

## Supplementary Material

Supplementary Information

## Figures and Tables

**Figure 1:**
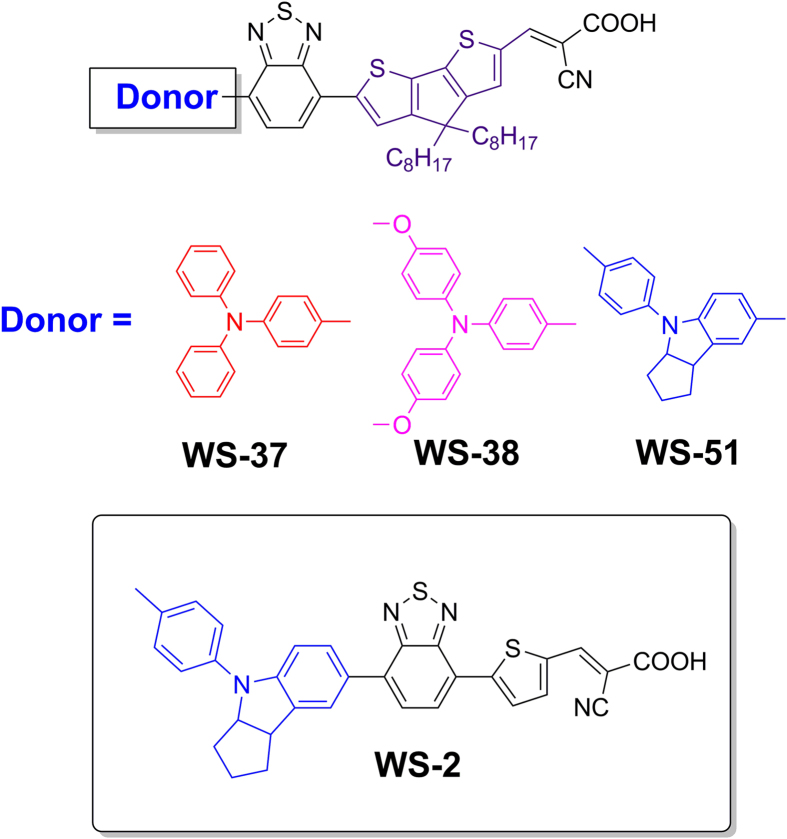
Molecular structures of CPDT-bridged D-A-π-A sensitizers WS-37, WS-38 and WS-51 derived from reference dye WS-2 .

**Table 1:** Absorption and electrochemical properties of sensitizers WS-37, WS-38 and WS-51 and reference dye WS-2.

**Dye**	***λ***_**max**_ **[nm]**^a^	***ε*** **[M**^**−1**^ **cm**^**−1**^]^a^	***λ***_**max**_**on TiO**_**2**_ **[nm]**^b^	**HOMO [*****V***]^c^	***E***_**0-0**_ **[e*****V***]^d^	**LUMO [*****V***]^d^
**WS-2**	533	16700	515	0.90	1.90	–1.00
	395	14100				
	321	21100				
**WS-37**	536	37300	514	1.05	1.96	–0.91
	457	16700				
	308	20900				
**WS-38**	546	41000	529	0.85	1.89	–1.04
	459	17200				
	308	22700				
**WS-51**	551	43000	533	0.82	1.83	–1.01
	460	27800				
	314	22700				

^a^Absorption peaks (*λ*_max_) and molar extinction coefficients (*ε*) in mixed solution (CHCl_3_:CH_3_OH, v/v = 4:1).

^b^Absorption peaks on TiO_2_ films.

^c^HOMO were measured in CH_2_Cl_2_ with 0.1 M tetrabutylammonium hexafluorophosphate (TBAPF_6_) as electrolyte (working electrode: Pt; reference electrode: SCE; calibrated with ferrocene/ferrocenium (Fc/Fc^+^) as an external reference. Counter electrode: Pt, and the scan rate is 100 mV/s.

^d^*E*_0-0_ was estimated from the absorption thresholds from absorption spectra of dyes adsorbed on the TiO_2_ film, LUMO is estimated by subtracting *E*_0-0_ from the HOMO.

**Table 2:** Photovoltaic parameters of DSSCs measured under AM 1.5 conditions (dye bath solvent: CHCl_3_:C_2_H_5_OH, v/v = 1:1, dipping time: 12 h).

**Dye**	***J***_**SC**_/**mA cm**^**−2**^	***V***_**OC**_/**mV**	***FF***	***η***
**WS-2**	18.24 ± 0.24	649 ± 5	0.718 ± 0.003	8.49 ± 0.13
**WS-37**	16.23 ± 0.31	692 ± 4	0.716 ± 0.002	8.04 ± 0.12
**WS-38**	12.32 ± 0.25	699 ± 5	0.727 ± 0.004	6.27 ± 0.12
**WS-51**	19.69 ± 0.09	700 ± 4	0.731 ± 0.002	10.08 ± 0.05
**WS-51 (5 mM CDCA)**	18.85 ± 0.12	687 ± 3	0.718 ± 0.001	9.29 ± 0.04

**Table 3:** Photovoltaic parameters of WS-2 and WS-51 measured with a transitory dye adsorption obtained from the averaged four devices (dye bath solvent of CH_2_Cl_2 _).

**Dye/dipping time**	***J***_**SC**_**/mA cm**^**−2**^	***V***_**OC**_**/mV**	***FF***	***η***
**WS-2**/1 h	16.09 ± 0.28	608 ± 3	0.703 ± 0.002	6.88 ± 0.11
**WS-2**/2 h	17.67 ± 0.23	617 ± 4	0.693 ± 0.003	7.63 ± 0.09
**WS-2**/12 h	12.43 ± 0.36	628 ± 6	0.704 ± 0.003	5.50 ± 0.17
**WS-51**/1 h	17.89 ± 0.15	637 ± 6	0.688 ± 0.006	7.84 ± 0.16
**WS-51**/2 h	19.01 ± 0.22	705 ± 3	0.673 ± 0.002	9.02 ± 0.03
**WS-51**/12 h	19.85 ± 0.08	683 ± 6	0.704 ± 0.003	9.55 ± 0.07
