# Supplementary material for: Rational molecular engineering of cyclopentadithiophene-bridged D-A-π-A sensitizers combining high photovoltaic efficiency with rapid dye adsorption
Source: Sci Rep. 2015 Jun 11;5:11330. doi: 10.1038/srep11330 (PMC4464146; doi:10.1038/srep11330)
Supplement: Supplementary Information [file srep11330-s1.pdf]

Supplementary Information

Rational molecular engineering of cyclopentadithiophene-bridged D-A-π-A sensitizers combining high photovoltaic efficiency with rapid dye adsorption

Qipeng Chai[1], Wenqin Li[1,2], Jingchuan Liu[1], Zhiyuan Geng[3], He Tian[1], and Wei-Hong Zhu[1]*

[1]Shanghai Key Laboratory of Functional Materials Chemistry, Key Laboratory for Advanced Materials and Institute of Fine Chemicals, Collaborative Innovation Center for Coal Based Energy (i-CCE), East China University of Science and Technology, Shanghai 200237, P. R. China, Fax: (+86) 21-6425-2758. E-mail: whzhu@ecust.edu.cn

[2]School of Urban Development and Environmental Engineering, Shanghai Second Polytechnic University, Shanghai 201209, P. R. China

[3]Gansu Key Laboratory of Polymer Materials, College of Chemistry and Chemical Engineering, Key Laboratory of Eco-environment-related Polymer Materials, Ministry of Education, Northwest Normal University, Lanzhou, 730070 Gansu, P. R. China

Contents

1. Calculated TDDFT (CAMB3LYP) excitation energies for the lowest transition (eV, nm), oscillator strengths (*f*), composition in terms of molecular orbital contributions, and experimental absorption maxima.
2. Cyclic voltammograms of reference dye WS-2 (a), WS-37, WS-38, and WS-51 (b).
3. Optimized ground-state geometries of sensitizers for WS-37, WS-38, and WS-51 and reference dye WS-2.
4. FTIR spectra of the TiO_2 films adsorbed by WS-2 (a) and WS-51 (b).
5. Materials and detailed synthetic procedure.
6. ^{1}H, ^{13}C NMR and H RMS spectra.

1. Calculated TDDFT (CAMB3LYP) excitation energies for the lowest transition (eV, nm), oscillator strengths (*f*), composition in terms of molecular orbital contributions, and experimental absorption maxima.

Table S1. Calculated TDDFT (CAMB3LYP) excitation energies for the lowest transition (eV, nm), oscillator strengths (*f*), composition in terms of molecular orbital contributions, and experimental absorption maxima.

Dye	State	Composition	*E*(eV, nm)	*f*	Exp.
**WS-2**	S1	H→L (83%)	2.34(530.6)	1.3278	1.90(533)
	S2	H-1→L (73%)	3.21(386.4)	0.2028	(395)
**WS-37**	S1	H→L (77%)	2.31(537.9)	1.9119	1.96(536)
	S3	H→L+2 (59%)	4.23(293.4)	0.3530	308
**WS-38**	S1	H→L (69%)	2.26(549.6)	1.9039	1.89(546)
	S3	H→L+2 (53%)	4.18(296.7)	0.3884	308
**WS-51**	S1	H→L (81%)	2.20(564.1)	1.9201	1.83(551)
	S2	H-1→L (41%)	2.86(432.9)	0.2013	460
		H→L+1 (47%)			
	S3	H→L+2 (61%)	4.20(295.1)	0.4319	314

2. Cyclic voltammograms of reference dye WS-2 (a), WS-37, WS-38, and WS-51 (b).

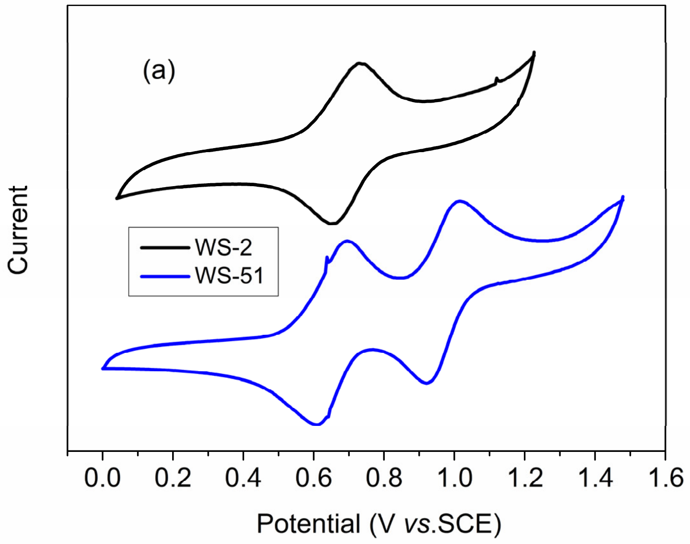

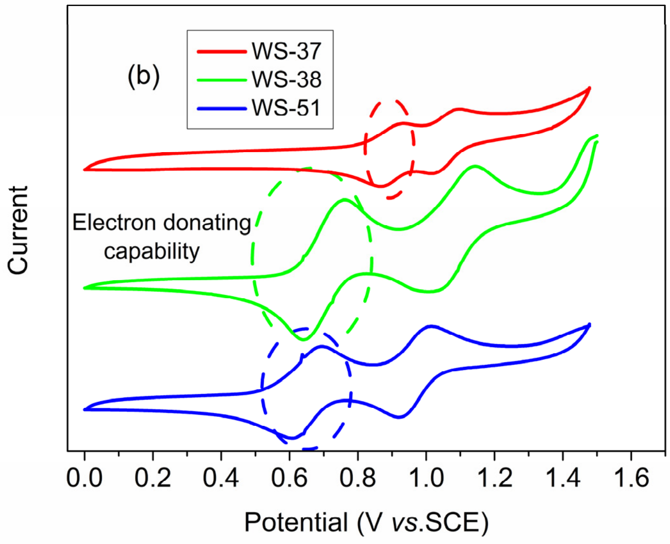

Figure S1. Cyclic voltammograms of reference dye **WS-2** (a), **WS-37**, **WS-38**, and **WS-51** (b) measured in CH_2Cl_2 solutions.

3. Optimized ground-state geometries of sensitizers for WS-37, WS-38, and WS-51 and reference dye WS-2.

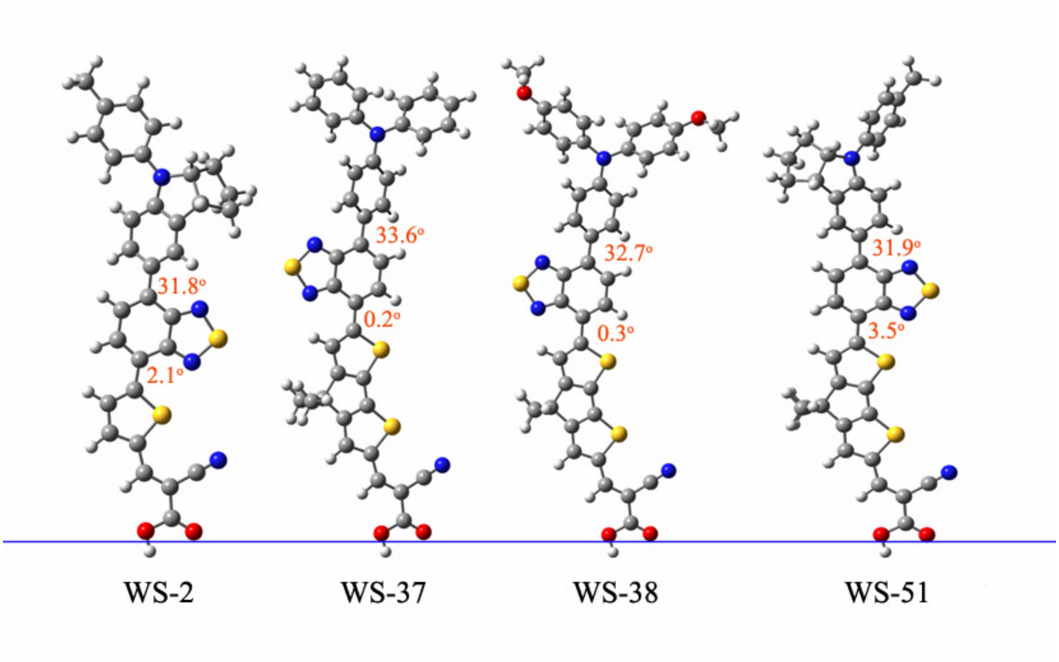

Figure S2. Optimized ground-state geometries of sensitizers for **WS-37**, **WS-38**, and **WS-51** and reference dye **WS-2**.

4. FTIR spectra of the TiO_2 films adsorbed by WS-2 (a) and WS-51 (b).

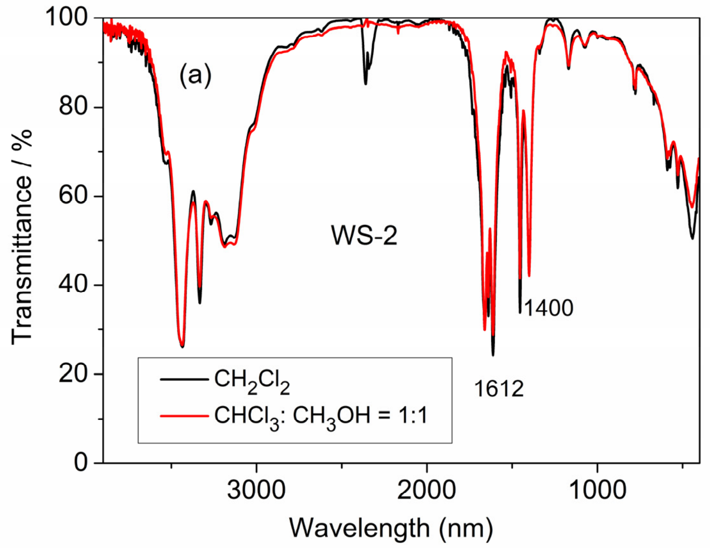

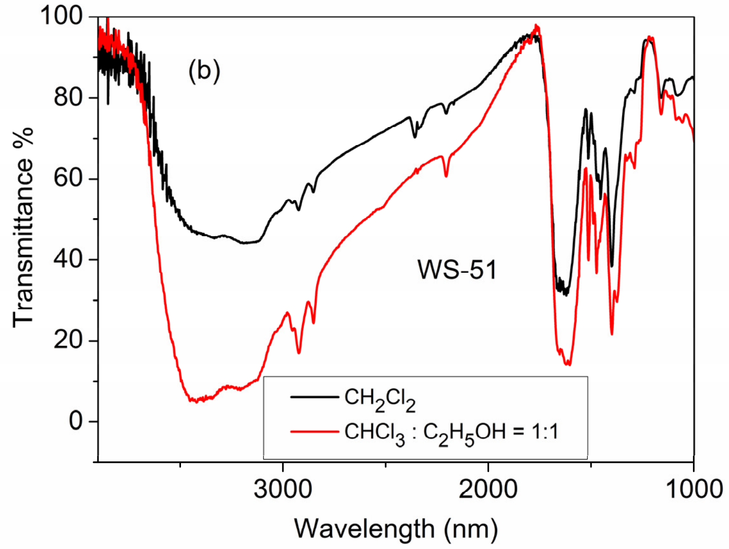

Figure S3. FTIR spectra of the TiO_2 films adsorbed by **WS-2** (a) and **WS-5**1 (b).

5. Materials and detailed synthetic procedure

Materials. The regents used in the synthesis procedure were all purchased with analytic grade, n-BuLi and tetrakis(triphenylphosphine)palladium(0) were from *J&K*, trimethyl borate was obtained from Adamas, and used without further treatment. THF was distilled from sodium for use until the indicator benzophenone turned blue. DMF was freshly distilled from CaH_2 and obtained under reduced pressure. All the reactions were conducted under argon atmosphere.

Synthesis of 2.

A mixture of compound **1** (2.0 g, 6.85 mmol), $Pd(PPh_3)_4$ (100 mg, 0.087 mmol), K_2CO_3 (2 M, 10 mL) were dissolved in THF (40 mL) and heated to reflux under argon atmosphere for 30 min. The dioctyl-substituted CPDT boric acid ester prepared previously was added dropwise, and then allowed to react for further 8 h. After cooling to ambient temperature, the mixture was washed with water, extracted with CH_2Cl_2 (50 mL × 3), and dried over anhydrous Na_2SO_4. The collected organic layer was evaporated, and the residue was purified by column chromatography on silica (petroleum ether : CH_2Cl_2 = 10:1) to give a red oil (1.5 g, yield 36%). ^{1}H NMR (400 MHz, $CDCl_3$, ppm): δ 8.03 (s, 1H), 7.82 (d, J = 8.0 Hz, 1H), 7.70 (d, J = 7.6 Hz, 1H), 7.26 (d, J = 4.8 Hz, 1H), 6.98 (d, J = 4.8 Hz, 1H), 1.88−1.92 (m, 4H), 1.15−1.22 (m, 20H), 1.00−1.02 (m, 4H), 0.82 (t, J = 7.2 Hz, 6H). ^{13}C NMR (100 MHz, $CDCl_3$, ppm): δ 159.15, 159.02, 153.90, 151.62, 139.25, 138.21, 136.36, 132.40, 128.11, 126.18, 124.05, 122.76, 121.77, 110.89, 53.85, 37.77, 31.82, 30.02, 29.35, 29.25, 24.59, 22.62, 14.09. HRMS (ESI, *m/z*): $[M + H]^+$ calcd for $C_{31}H_{40}BrN_2S_3$: 615.1537; found: 615.1517.

Synthesis of 3.

$POCl_3$ (1.1 g, 7.17 mmol) was added dropwise to a solution of anhydrous DMF (20 mL) at 0 ºC, and then stirred for 30 min. **2** (1.5 g, 2.43 mmol) dissolved in anhydrous DMF (10 mL) was added slowly, and reacted for further 12 h under ambient temperature. The mixture was poured into a saturated aqueous solution of CH_3COOK and stirred for another 30 min. Then it was extracted with CH_2Cl_2 (50 mL × 3) and dried over anhydrous Na_2SO_4. After removal of the solvent, the residue was purified by column chromatography on silica (petroleum ether : CH_2Cl_2 = 2:1) to give an orange oil (1.2 g, yield 77%). ^{1}H NMR (400 MHz, $CDCl_3$, ppm): δ 9.87 (s, 1H), 8.04 (s, 1H), 7.88 (d, J = 7.6 Hz, 1H), 7.77 (d, J = 8.0 Hz, 1H), 7.61 (s, 1H), 1.93−1.97 (m, 4H), 1.16−1.25 (m, 20 H), 0.97−1.01 (m, 4H), 0.82 (t, J = 6.8 Hz, 6H). ^{13}C NMR (100 MHz, $CDCl_3$, ppm): δ 182.67, 162.94, 158.62, 153.88, 151.47, 147.19, 144.10, 143.02, 137.59, 132.30, 127.24, 125.19, 122.37, 112.59, 54.30, 37.65, 31.77, 29.91, 29.70, 29.29, 29.21, 24.63, 22.59, 14.07. HRMS (ESI, *m/z*): $[M + H]^+$ calcd for $C_{32}H_{40}BrN_2OS_3$: 643.1486; found: 643.1489.

Synthesis of 4a.

A mixture of compound **3** (300 mg, 0.47 mmol), $Pd(PPh_3)_4$ (30 mg, 0.03 mmol), K_2CO_3 (2 M, 10 mL), THF (50 mL) was stirred and heated to reflux for 30 min under argon atmosphere. The 4-substituted boric acid ester of triphenylamine synthesized previously was added dropwise and refluxed for another 8 h. After cooling to ambient temperature, then the mixture was poured into water, extracted with CH_2Cl_2 (50 mL × 3), and dried over anhydrous Na_2SO_4. The collected organic layer was evaporated, and the residue was purified by column chromatography on silica

(petroleum ether : CH_2Cl_2 =1:1) to give a red oil (240 mg, yield 64%). ^{1}H NMR (400 MHz, $CDCl_3$, ppm): δ 9.86 (s, 1H), 8.06 (s, 1H), 7.99 (d, J = 7.6 Hz, 1H), 7.90 (d, J = 8.8 Hz, 2H), 7.73 (d, J = 7.6 Hz, 1H), 7.60 (s, 1H), 7.29−7.32 (m, 4H), 7.19−7.22 (m, 6H), 7.06−7.10 (m, 2H), 1.94−1.98 (m, 4H), 1.16−1.23 (m, 20H), 0.97−1.02 (m, 4H), 0.82 (t, J = 6.8 Hz, 6H). ^{13}C NMR (100 MHz, $CDCl_3$, ppm): δ 182.59, 163.06, 158.37, 153.97, 152.57, 148.27, 147.71, 147.37, 144.39, 143.71, 136.92, 132.67, 130.43, 129.92, 129.41, 127.11, 125.79, 125.50, 125.01, 123.47, 122.72, 121.60, 54. 26, 37.73, 31.80, 29.97, 29.32, 29.23, 24.66, 22.61, 14.08. HRMS (ESI, *m/z*): $[M + H]^+$ calcd for $C_{50}H_{54}N_3OS_3$: 808.3429; found: 808.3435.

Synthesis of 4b.

4b was obtained as a deep red oil in a way similar to that for **4a** (180 mg, yield 58%). ^{1}H NMR (400 MHz, $CDCl_3$, ppm): δ 9.86 (s, 1H), 8.05 (s, 1H), 7.97 (d, J = 7.6 Hz, 1H), 7.84 (d, J = 8.8 Hz, 2H), 7.69 (d, J = 7.6 Hz, 1H), 7.60 (s, 1H), 7.05−7.16 (m, 6H), 6.87 (d, J = 8.8 Hz, 4H), 3.82 (s, 6H), 1.94−1.98 (m, 4H), 1.16−1.22 (m, 20H), 0.99−1.03 (m, 4H), 0.82 (t, J = 7.2 Hz, 6H). ^{13}C NMR (100 MHz, $CDCl_3$, ppm): δ 182.59, 163.07, 158.31, 156.27, 154.02, 152.60, 149.16, 147.79, 144.54, 143.62, 140.40, 136.75, 132.96, 129.78, 128.43, 127.14, 126.67, 126.39, 125.62, 125.38, 121.42, 119.59, 114.81, 55.53, 54.24, 37.73, 31.80, 29.97, 29.72, 29.24, 24.66, 22.62, 14.09. HRMS (ESI, *m/z*): $[M + H]^+$ calcd for $C_{52}H_{58}N_3O_3S_3$: 868.3640; found: 868.3641.

Synthesis of 4c.

4c was obtained as a purple solid in a way similar to that for **4a** (220 mg, yield 56%).
^{1}H NMR (400 MHz, $CDCl_3$, ppm): δ 9.85 (s, 1H), 8.04 (s, 1H), 7.96 (d, J = 8.0 Hz, 1H), 7.80 (s, 1H), 7.75 (d, J = 8.4 Hz, 1H), 7.69 (d, J = 7.6 Hz, 1H), 7.60 (s, 1H), 7.25 (d, J = 8.0 Hz, 2H), 7.19 (d, J = 8.0 Hz, 2H), 7.03 (d, J = 8.0 Hz, 1H), 4.86−4.90 (m, 1H), 3.93−3.97 (m, 1H), 2.35 (s, 3H), 2.05−2.16 (m, 1H), 1.93−1.98 (m, 6H), 1.77−1.87 (m, 1H), 1.65−1.72 (m, 1H), 1.55−1.64 (m, 1H), 1.12−1.27 (m, 20H), 0.97−1.06 (m, 4H), 0.82 (t, J = 6.8 Hz, 6H). ^{13}C NMR (100 MHz, $CDCl_3$, ppm): δ 182.56, 163.11, 158.23, 154.12, 152.66, 148.58, 147.89, 144.78, 143.52, 140.04, 136.51, 135.49, 133.70, 131.90, 129.85, 128.94, 127.06, 126.05, 125.76, 125.54, 124.77, 121.18, 120.42, 107.54, 69.43, 54.22, 45.45, 37.74, 35.23, 33.68, 31.81, 29.98, 29.33, 29.25, 24.67, 24.48, 22.63, 20.86, 14.10. HRMS (ESI, *m/z*): $[M + H]^+$ calcd for $C_{50}H_{58}N_3OS_3$: 812.3742; found: 812.3746.

Synthesis of WS-37.

The precursor **4a** (180 mg, 0.22 mmol) and cyanoacetic acid (187 mg, 2.2 mmol) were mixed together and dissolved in 20 mL acetonitrile with 0.5 mL piperidine as catalyst. Then the mixture was stirred under argon atmosphere and heated to reflux for 8 h. After cooling to ambient temperature, it was washed with water, extracted with CH_2Cl_2 (50 mL × 3), and dried over anhydrous Na_2SO_4. The collected organic layer was removed by evaporation, and the residue was purified by column chromatography on silica (CH_2Cl_2 / MeOH = 10/1) to give a purple solid (140 mg, yield 69%). ^{1}H NMR (400 MHz, DMSO-d_6, ppm): δ 8.27 (s, 2H), 8.22 (d, J =8.0 Hz, 1H), 8.00 (d, J = 8.8 Hz, 2H), 7.91 (d, J = 7.6 Hz, 1H), 7.84 (s, 1H), 7.35−7.39 (m, 4H), 7.10−7.13 (m, 8H), 1.94−1.99 (m, 4H), 1.11−1.23 (m, 24H), 0.75 (t, J = 6.8 Hz, 6H). ^{13}C NMR (100 MHz, THF-d_8, ppm): δ 162.22, 158.88, 153.63, 152.30, 147.98, 147.52, 145.31, 143.97, 138.24, 137.50, 131.66, 130.47, 129.84, 129.22, 126.57, 125.67, 124.76, 124.46, 123.19, 122.35, 121.89, 54.16, 37.67, 31.87, 30.13, 29.43, 29.37, 23.95, 22.55, 13.50. HRMS (ESI, *m/z*): $[M + H]^+$ calcd for

$C_{53}H_{55}N_4O_2S_3$: 875.3487; found: 875.3479.

Synthesis of WS-38.

WS-38 was obtained as a purple black solid according to the above mentioned procedure (104 mg, yield 72%). ^{1}HNMR (400 MHz, DMSO-d_6, ppm): δ 8.33 (s, 1H), 8.19 (s, 1H), 8.03 (s, 1H), 7.71−7.82 (m, 4H), 7.07 (d, J = 8.8 Hz, 4H), 6.93 (d, J = 8.8 Hz, 4H), 6.85 (d, J = 8.8 Hz, 2H), 3.75 (s, 6H), 1.89−1.92 (m, 4H), 0.93−1.05 (m, 24H), 0.70 (t, J = 6.8 Hz, 6H). ^{13}C NMR (100 MHz, DMSO-d_6, ppm): δ 161.96, 158.14, 156.00, 152.98, 151.63, 148.53, 145.41, 143.37, 139.57, 137.37, 136.98, 131.36, 129.69, 127.55, 126.98, 126.53, 125.40, 124.45, 121.29, 118.39, 114.95, 55.20, 53.40, 36.73, 31.11, 29.16, 28.57, 28.50, 23.99, 21.97, 13.78. HRMS (ESI, *m/z*): $[M + H]^+$ calcd for $C_{55}H_{59}N_4O_4S_3$: 935.3698; found: 935.3693.

Synthesis of WS-51.

WS-51 was obtained as a purple black solid according to the above mentioned procedure (150 mg, 63%). ^{1}H NMR (400 MHz, THF-d_8, ppm): δ 8.40−8.68 (m, 1H), 8.24 (s, 1H), 7.59−8.00 (m, 4H), 7.42−7.52 (m, 1H), 7.15−7.22 (m, 4H), 6.88−6.95 (m, 1H), 4.83−4.91 (m, 1H), 3.86−3.90 (m, 1H), 2.31 (s, 3H), 1.98−2.12 (m, 4H), 1.85−1.95 (m, 3H), 1.62−1.66 (m, 1H), 1.47−1.56 (m, 2H), 0.98−1.29 (m, 24H), 0.74−0.85 (m, 6H). ^{13}C NMR (100 MHz, THF-d_8, ppm): δ 162.97, 158.43, 153.88, 152.61, 148.40, 148.08, 146.13, 145.35, 140.15, 137.31, 136.73, 135.11, 133.41, 131.37, 129.98, 129.57, 128.82, 127.08, 125.64, 125.46, 124.47, 124.25, 121.23, 120.11, 107.01, 69.11, 54.03, 45.35, 37.65, 35.13, 33.49, 31.76, 29.92, 29.24, 29.20, 24.83, 24.63, 22.48, 19.91, 13.42. HRMS (ESI, *m/z*): $[M–H]^-$ calcd for $C_{53}H_{57}N_4O_2S_3$: 877.3644; found: 877.3649.

6. ^{1}H, ^{13}C NMR and HRMS spectra

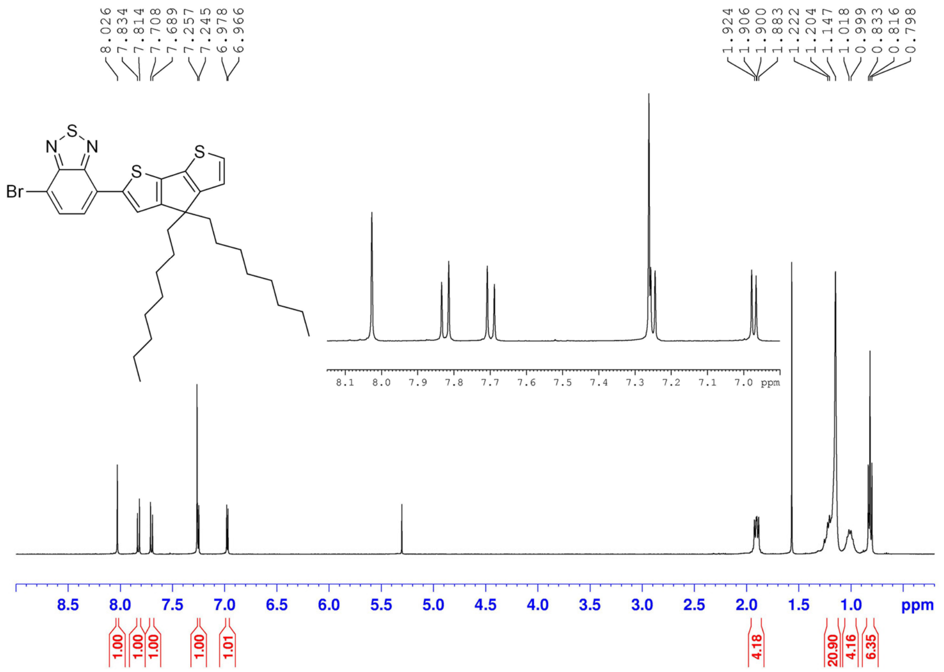

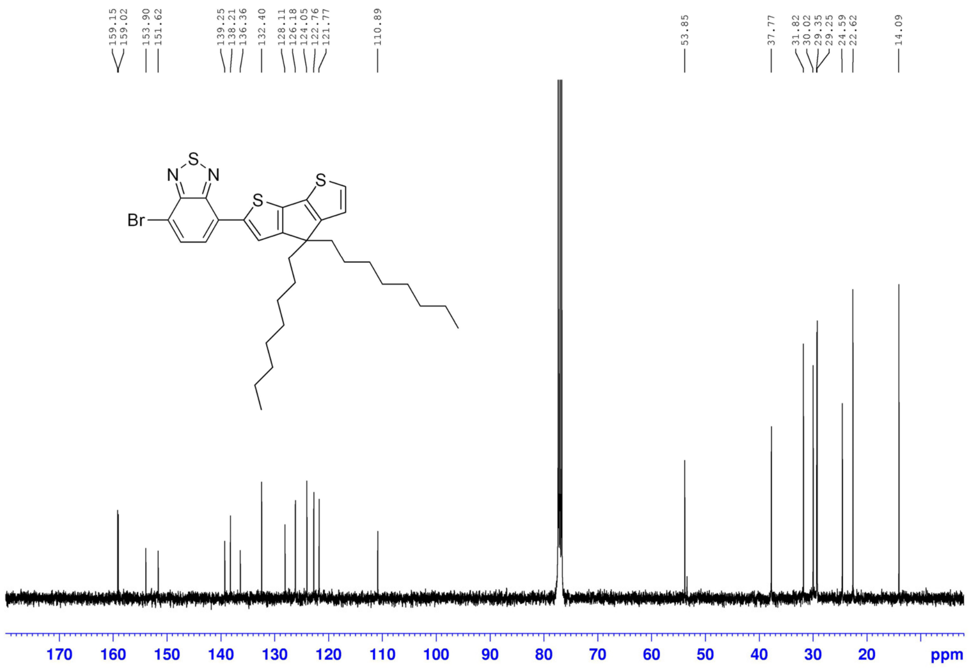

Figure S4. ^{1}H and ^{13}C NMR spectra of **2**.

Elemental Composition Report

Single Mass Analysis

Tolerance = 200.0 mDa / DBE: min = -1.5, max = 100.0
Element prediction: Off
Number of isotope peaks used for i-FIT = 2

Monoisotopic Mass, Even Electron Ions
27 formula(e) evaluated with 3 results within limits (up to 1 best isotopic matches for each mass)
Elements Used:
C: 0-31 H: 0-80 N: 0-2 S: 0-3 Br: 0-1

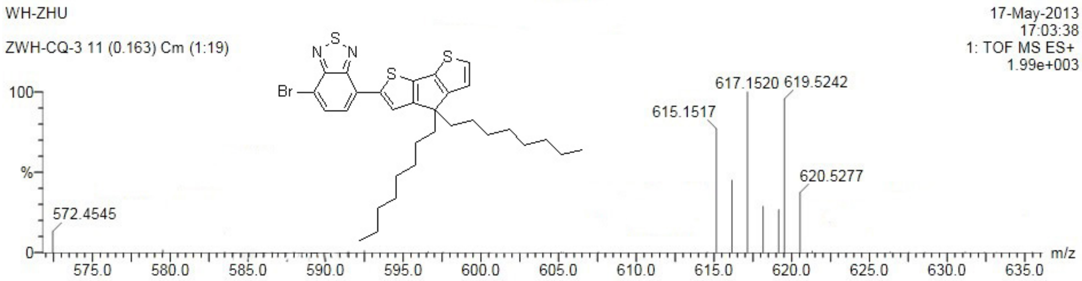

Minimum:				-1.5			
Maximum:		200.0	50.0	100.0			
Mass	Calc. Mass	mDa	PPM	DBE	i-FIT	i-FIT (Norm)	Formula
615.1517	615.1537	-2.0	-3.3	12.5	13.0	0.0	C31 H40 N2 S3 Br

Figure S5. HRMS spectrum of **2**.

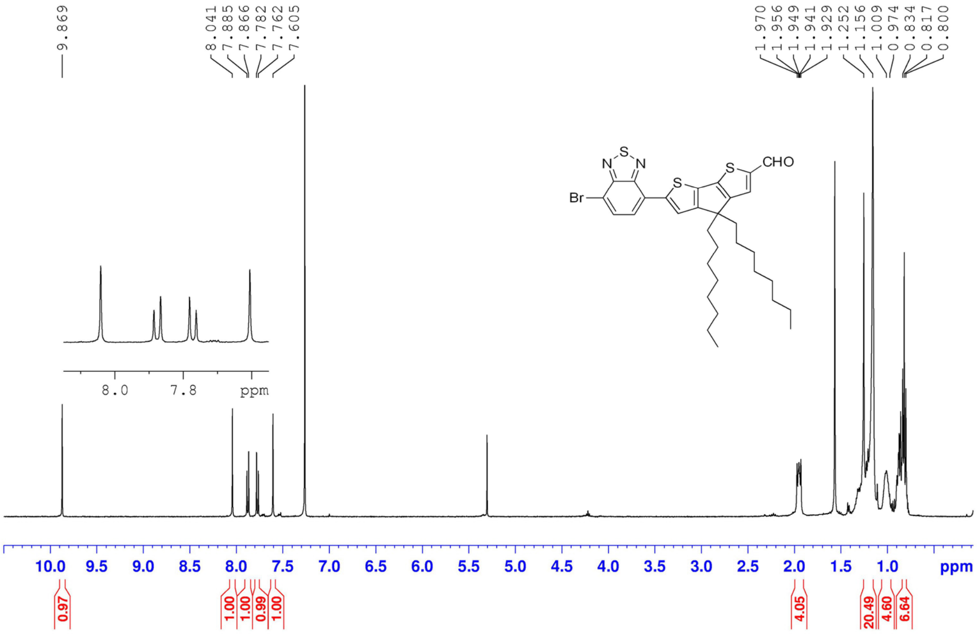

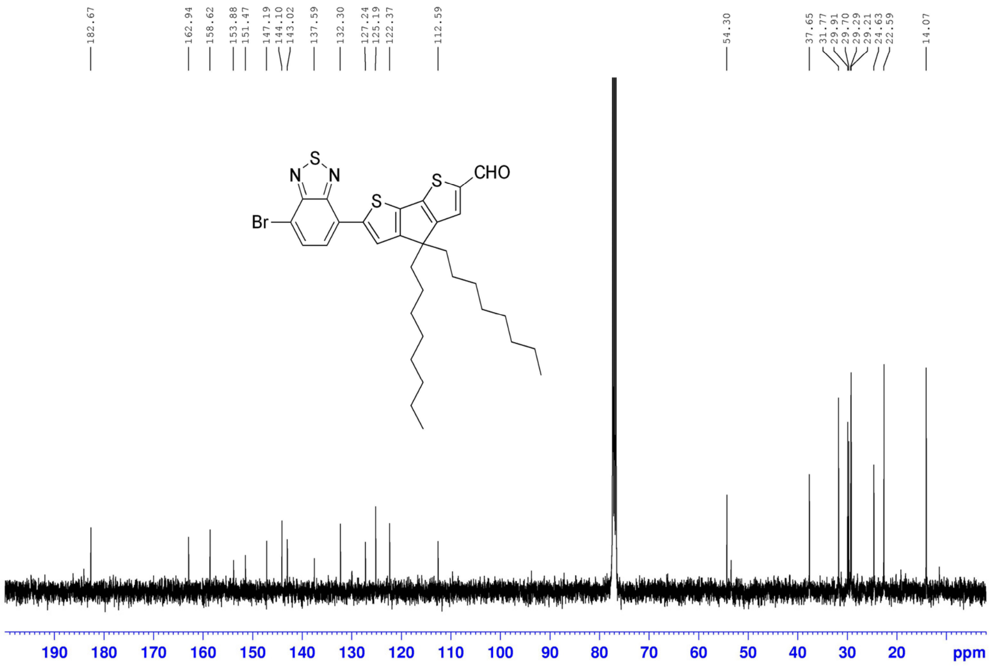

Figure S6. ^{1}H and ^{13}C NMR spectra of **3**.

Elemental Composition Report

Single Mass Analysis
Tolerance = 50.0 PPM / DBE: min = -1.5, max = 100.0
Element prediction: Off
Number of isotope peaks used for i-FIT = 2

Monoisotopic Mass, Even Electron Ions
209 formula(e) evaluated with 12 results within limits (up to 1 best isotopic matches for each mass)
Elements Used:
C: 0-32 H: 0-45 N: 0-2 O: 0-3 S: 0-3 Br: 0-2

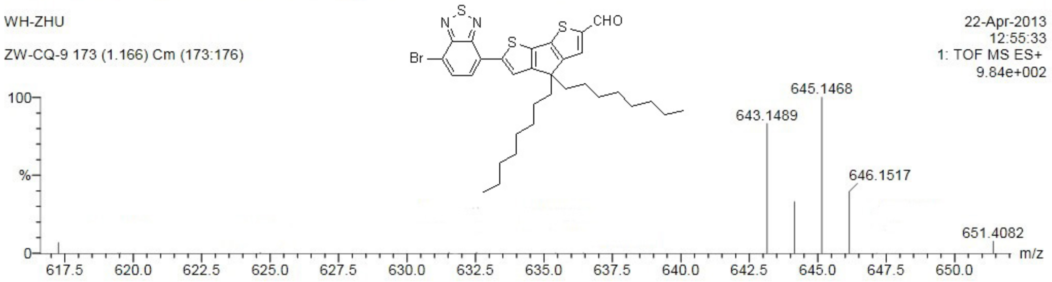

Minimum:				-1.5			
Maximum:		30.0	50.0	100.0			
Mass	Calc. Mass	mDa	PPM	DBE	i-FIT	i-FIT (Norm)	Formula
643.1489	643.1486	0.3	0.5	13.5	7.4	0.0	C32 H40 N2 O S3 Br

Figure S7. HRMS spectrum of **3**.

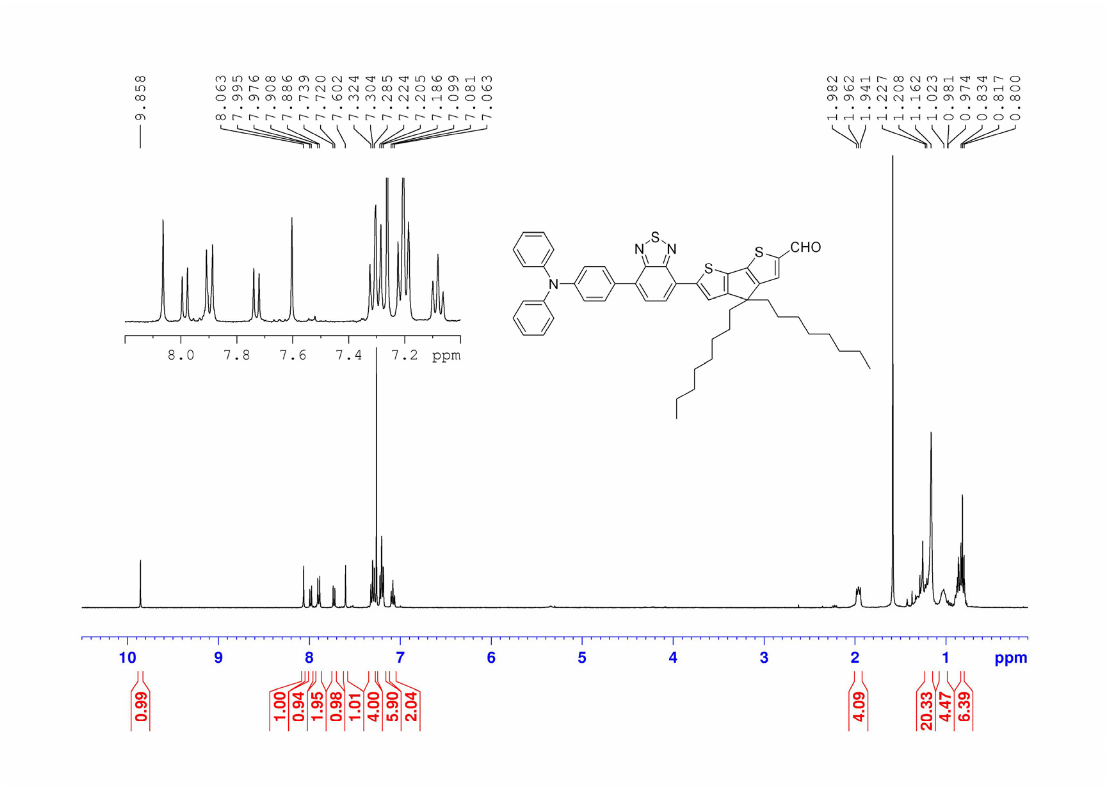

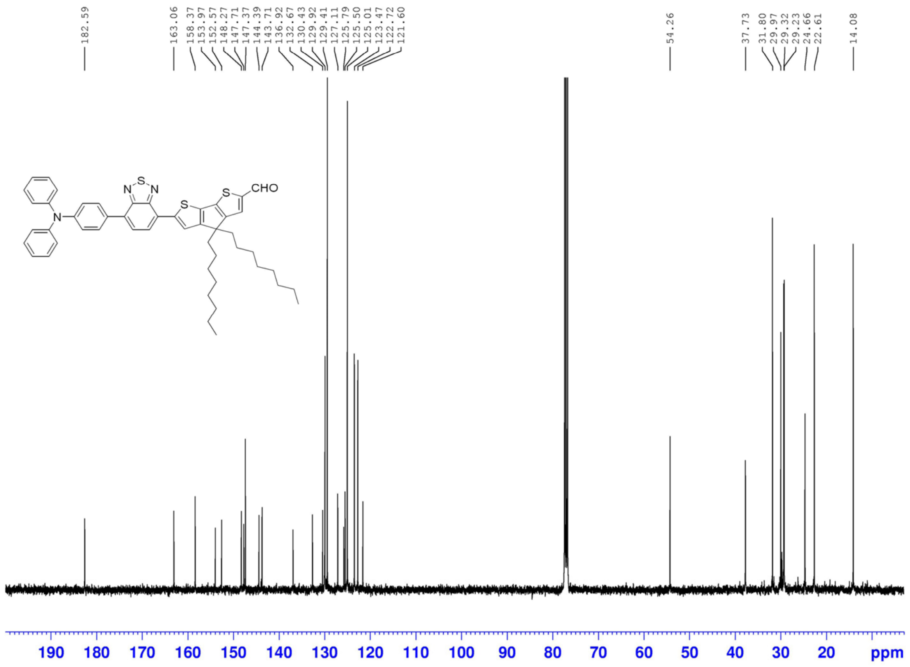

Figure S8. ^{1}H and ^{13}C NMR spectra of **4a**.

Elemental Composition Report

Single Mass Analysis
Tolerance = 50.0 PPM / DBE: min = -1.5, max = 100.0
Element prediction: Off
Number of isotope peaks used for i-FIT = 2

Monoisotopic Mass, Even Electron Ions
25 formula(e) evaluated with 1 results within limits (up to 1 best isotopic matches for each mass)
Elements Used:
C: 0-50 H: 0-75 N: 0-3 O: 0-1 S: 0-3
WH-ZHU

ZW-CQ-06 73 (0.535) Cm (67:73)

22-Apr-2013
19:02:59
1: TOF MS ES+
2.15e+003

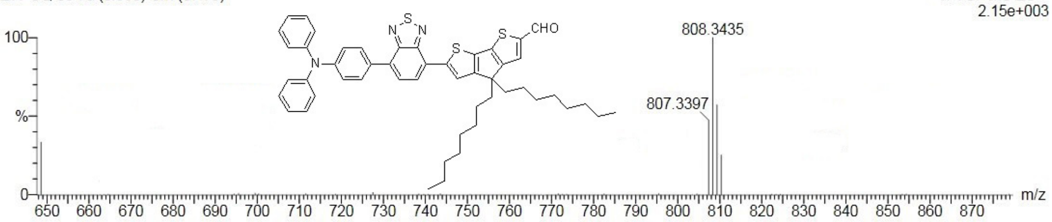

Mass	Calc. Mass	mDa	PPM	DBE	i-FIT	i-FIT (Norm)	Formula
Minimum:				-1.5			
Maximum:		30.0	50.0	100.0			
808.3435	808.3429	0.6	0.7	25.5	9.8	0.0	C50 H54 N3 O S3

Figure S9. HRMS spectrum of **4a**.

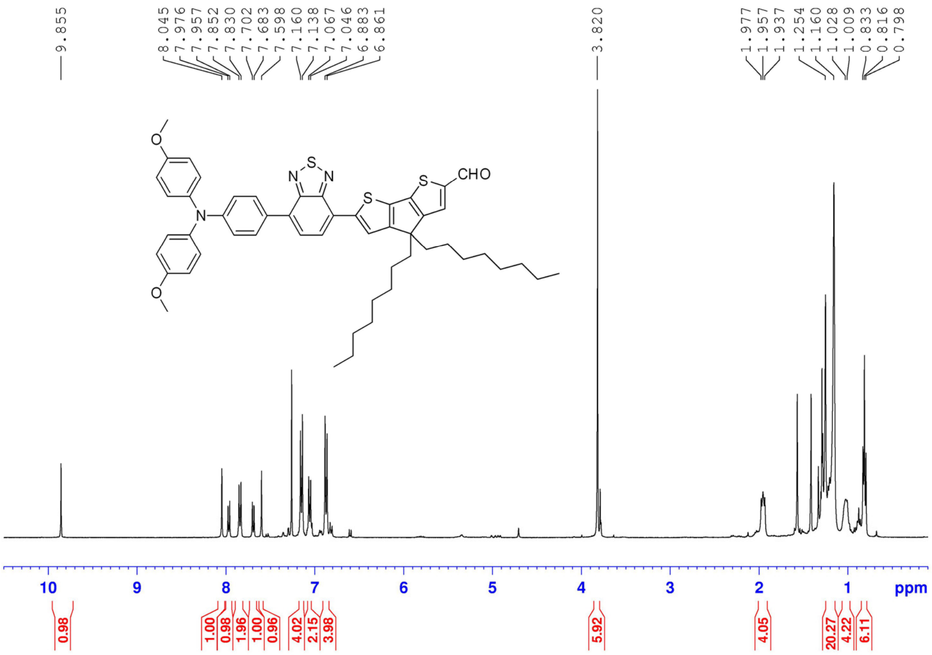

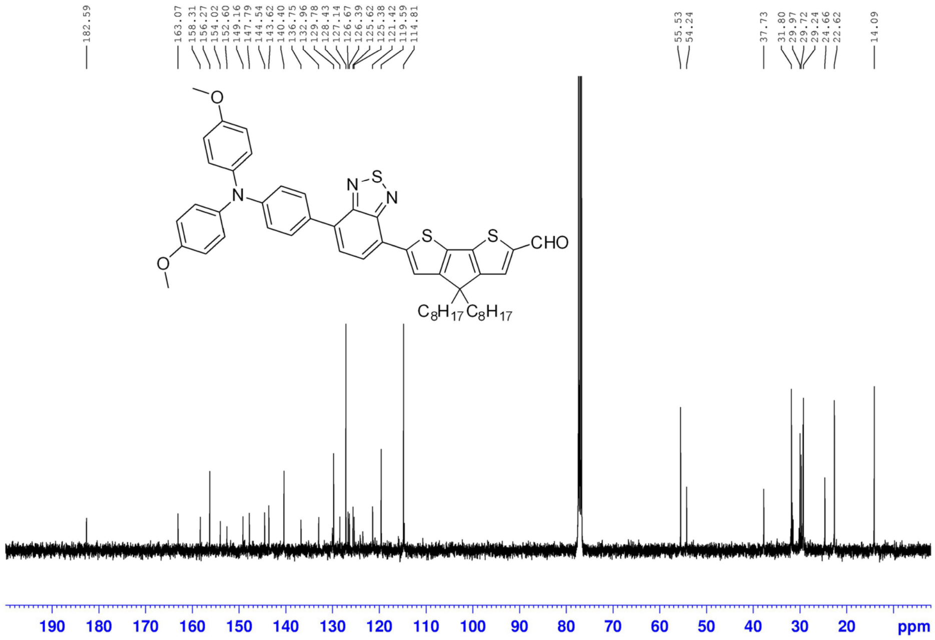

Figure S10. ^{1}H and ^{13}C NMR spectra of **4b**.

Elemental Composition Report

Single Mass Analysis

Tolerance = 30.0 mDa / DBE: min = -1.5, max = 100.0
Element prediction: Off
Number of isotope peaks used for i-FIT = 2

Monoisotopic Mass, Even Electron Ions
51 formula(e) evaluated with 1 results within limits (up to 1 closest results for each mass)
Elements Used:
C: 0-52 H: 0-60 N: 0-3 O: 0-3 S: 0-3
WH-ZHU

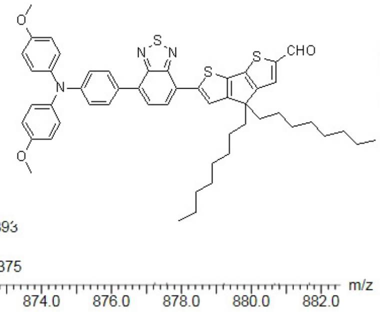

ZWH-CQ-4 50 (1.626) Cm (41:50)

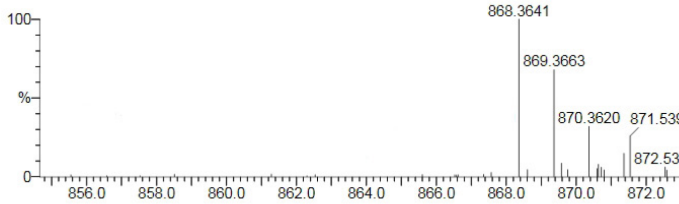

Minimum:				-1.5			
Maximum:		30.0	50.0	100.0			
Mass	Calc. Mass	mDa	PPM	DBE	i-FIT	i-FIT (Norm)	Formula
868.3641	868.3640	0.1	0.1	25.5	29.7	0.0	C52 H58 N3 O3 S3

Figure S11. HRMS spectrum of **4b**.

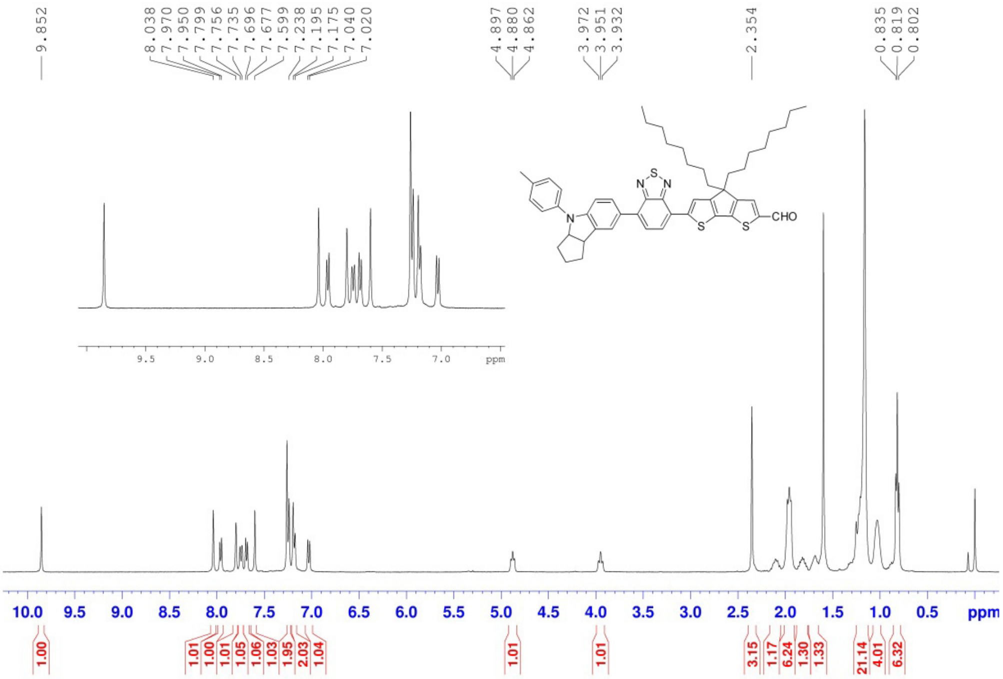

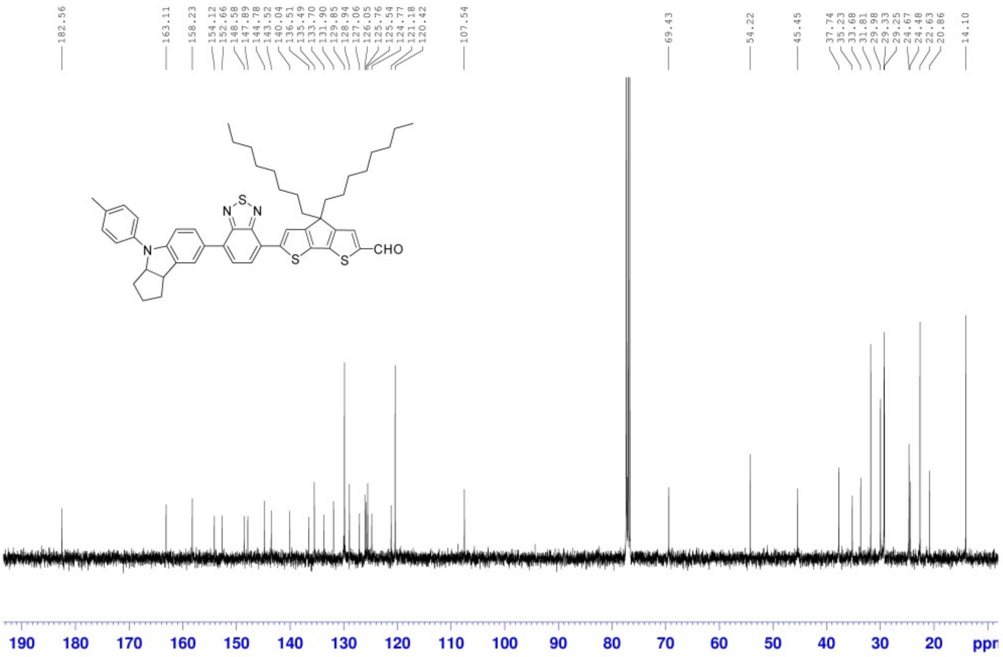

Figure S12. ^{1}H and ^{13}C NMR spectra of **4c**.

Elemental Composition Report **Page 1**

Single Mass Analysis
Tolerance = 30.0 mDa / DBE: min = -1.5, max = 100.0
Element prediction: Off
Number of isotope peaks used for i-FIT = 2

Monoisotopic Mass, Even Electron Ions
295 formula(e) evaluated with 19 results within limits (up to 1 closest results for each mass)
Elements Used:
C: 0-55 H: 0-60 N: 0-3 O: 0-6 S: 0-3

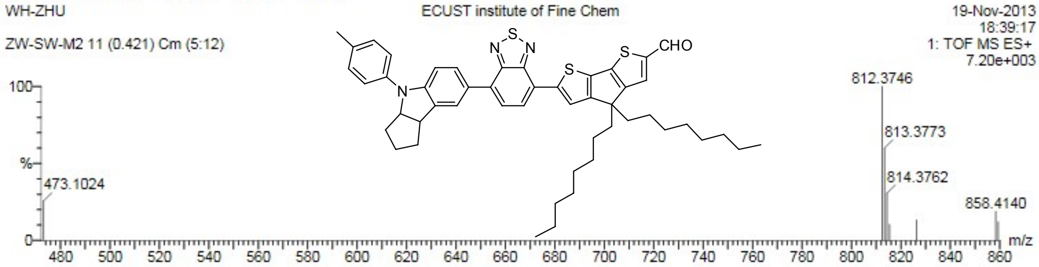

Minimum:				-1.5			
Maximum:		30.0	50.0	100.0			
Mass	Calc. Mass	mDa	PPM	DBE	i-FIT	i-FIT (Norm)	Formula
812.3746	812.3742	0.4	0.5	23.5	8.2	0.0	C50 H58 N3 O S3

Figure S13. HRMS spectrum of **4c**.

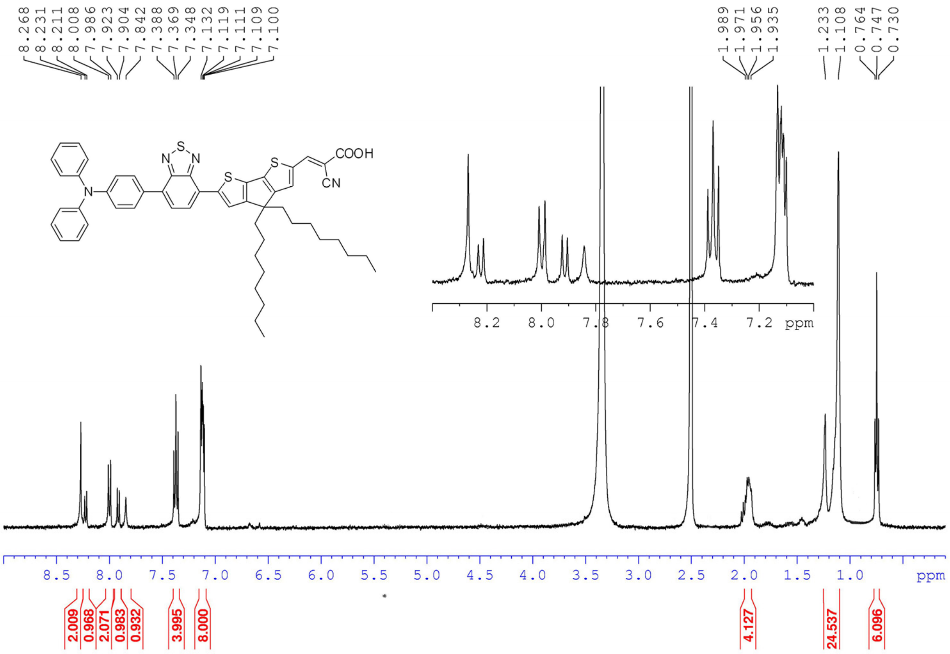

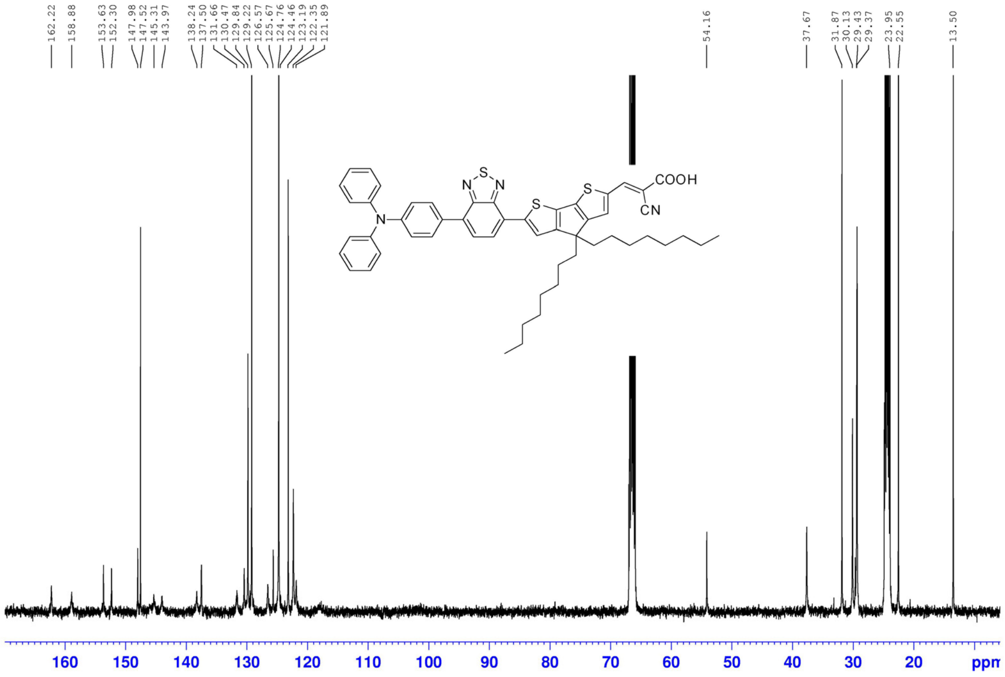

Figure S14. ^{1}H and ^{13}C NMR spectra of **WS-37**.

Elemental Composition Report

Single Mass Analysis
Tolerance = 200.0 mDa / DBE: min = -1.5, max = 100.0
Element prediction: Off
Number of isotope peaks used for i-FIT = 2

Monoisotopic Mass, Even Electron Ions
46 formula(e) evaluated with 4 results within limits (up to 1 best isotopic matches for each mass)
Elements Used:
C: 0-53 H: 0-80 N: 0-4 O: 0-2 S: 0-3

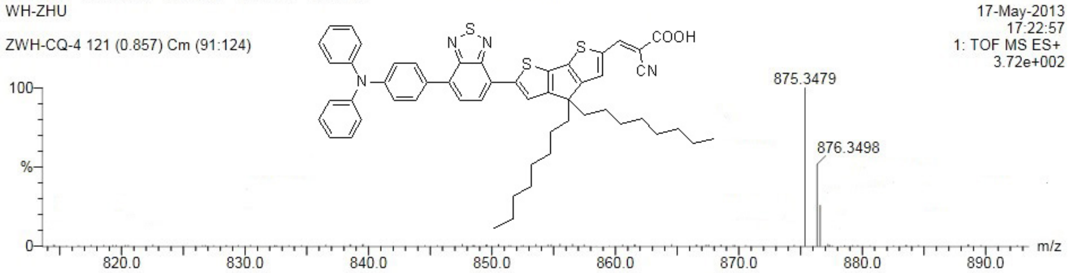

Minimum:				-1.5			
Maximum:		200.0	50.0	100.0			
Mass	Calc. Mass	mDa	PPM	DBE	i-FIT	i-FIT (Norm)	Formula
875.3479	875.3487	-0.8	-0.9	28.5	18.5	0.0	C53 H55 N4 O2 S3

Figure S15. HRMS spectrum of **WS-37**.

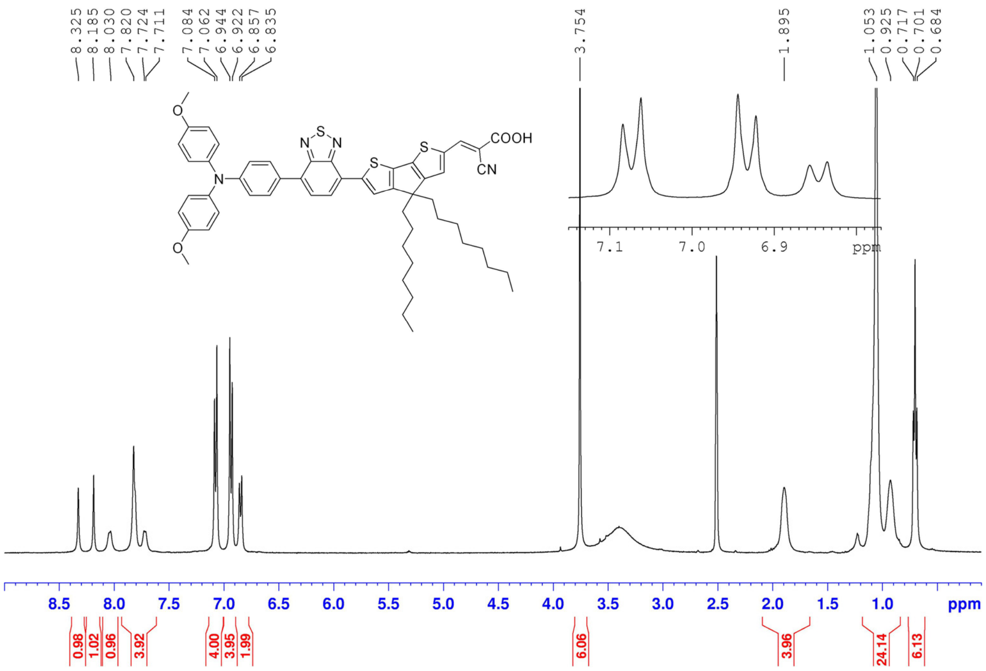

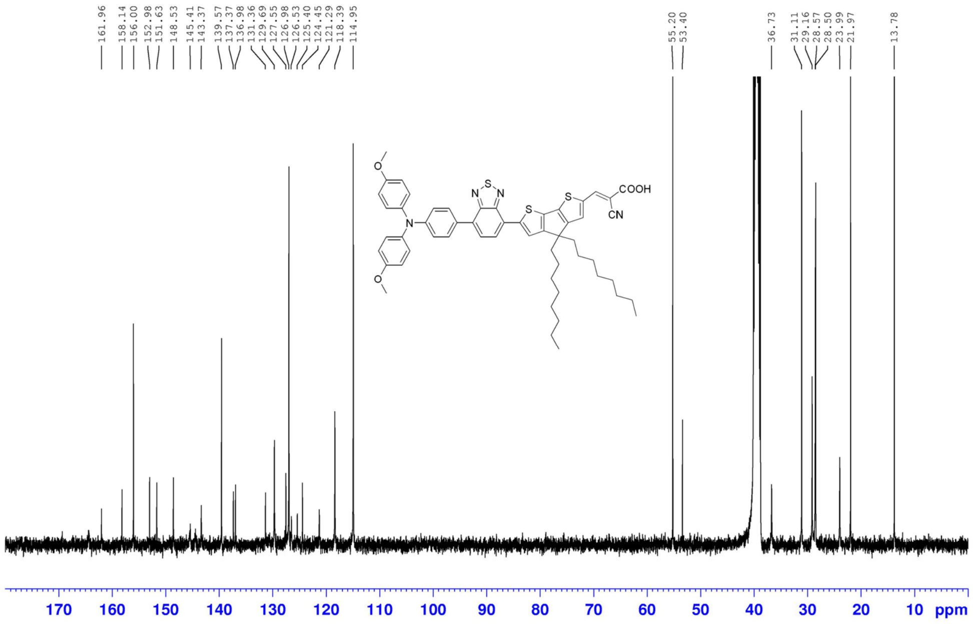

Figure S16. ^{1}H and ^{13}CNMR spectra of **WS-38**.

Elemental Composition Report

Single Mass Analysis
Tolerance = 30.0 mDa / DBE: min = -1.5, max = 100.0
Element prediction: Off
Number of isotope peaks used for i-FIT = 2

Monoisotopic Mass, Even Electron Ions
79 formula(e) evaluated with 1 results within limits (up to 1 best isotopic matches for each mass)
Elements Used:
C: 0-55 H: 0-60 N: 0-4 O: 0-4 S: 0-3

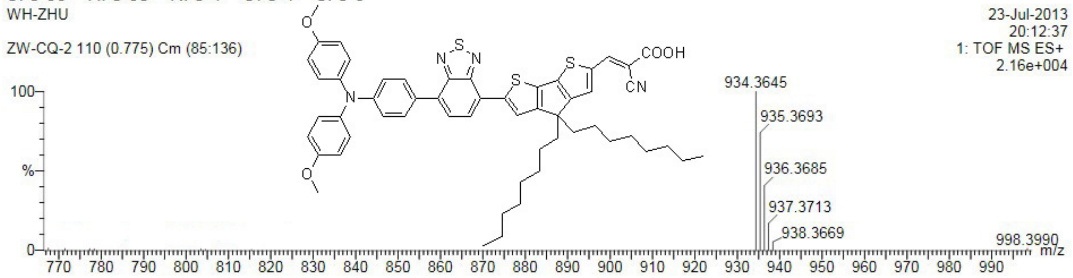

Minimum:				-1.5			
Maximum:		30.0	50.0	100.0			

Mass	Calc. Mass	mDa	PPM	DBE	i-FIT	i-FIT (Norm)	Formula
935.3693	935.3698	-0.5	-0.5	28.5	17.9	0.0	C55 H59 N4 O4 S3

Figure S17. HRMS spectrum of **WS-38**.

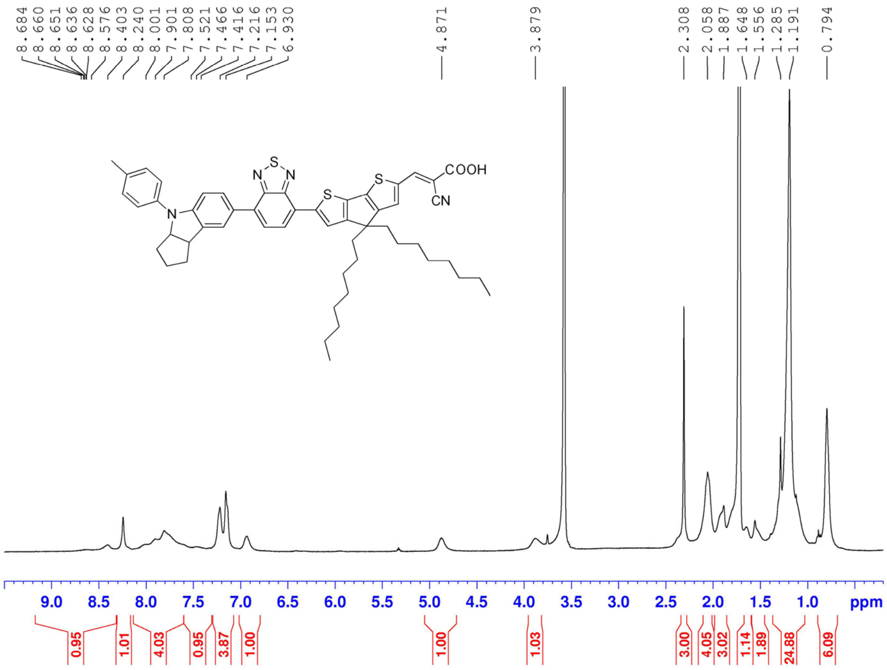

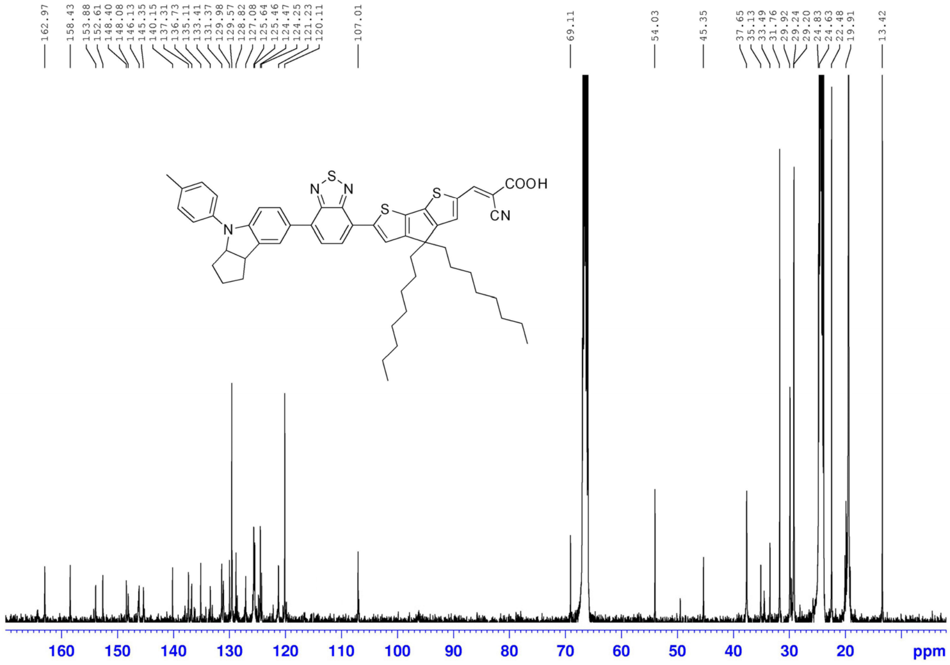

Figure S18. ^1H and ^{13}C NMR spectra of **WS-51**.

Elemental Composition Report

Single Mass Analysis
Tolerance = 30.0 mDa / DBE: min = -1.5, max = 100.0
Element prediction: Off
Number of isotope peaks used for i-FIT = 2

Monoisotopic Mass, Even Electron Ions
58 formula(e) evaluated with 1 results within limits (up to 1 closest results for each mass)
Elements Used:
C: 0-53 H: 0-100 N: 0-4 O: 0-2 S: 0-3
WH-ZHU

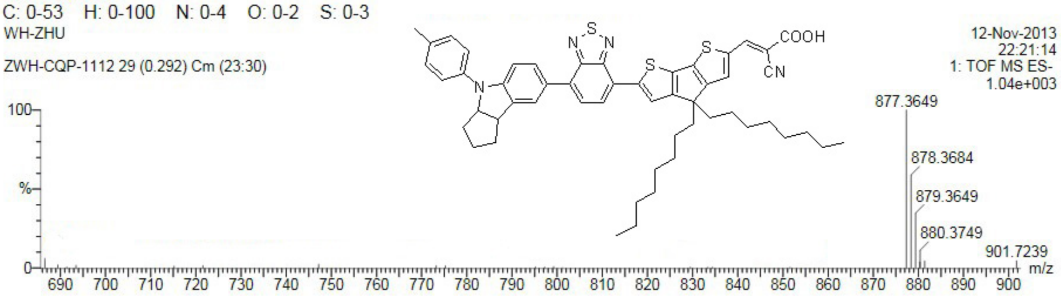

Minimum:				-1.5			
Maximum:		30.0	50.0	100.0			
Mass	Calc. Mass	mDa	PPM	DBE	i-FIT	i-FIT (Norm)	Formula
877.3649	877.3644	0.5	0.6	27.5	8.6	0.0	C53 H57 N4 O2 S3

Figure S19. HRMS spectrum of **WS-51**.
